# Super-Selective Reconstruction of Causal and Direct Connectivity With Application to *in vitro* iPSC Neuronal Networks

**DOI:** 10.3389/fnins.2021.647877

**Published:** 2021-07-16

**Authors:** Francesca Puppo, Deborah Pré, Anne G. Bang, Gabriel A. Silva

**Affiliations:** ^1^BioCircuits Institute and Center for Engineered Natural Intelligence, University of California, San Diego, La Jolla, CA, United States; ^2^Conrad Prebys Center for Chemical Genomics, Sanford Burnham Prebys Medical Discovery Institute, La Jolla, CA, United States; ^3^BioCircuits Institute, Center for Engineered Natural Intelligence, Department of Bioengineering, Department of Neurosciences, University of California, San Diego, La Jolla, CA, United States

**Keywords:** neuronal network, effective connectivity, functional connectivity, apparent connectivity, correlation, MEA, iPSC

## Abstract

Despite advancements in the development of cell-based *in-vitro* neuronal network models, the lack of appropriate computational tools limits their analyses. Methods aimed at deciphering the effective connections between neurons from extracellular spike recordings would increase utility of *in vitro* local neural circuits, especially for studies of human neural development and disease based on induced pluripotent stem cells (hiPSC). Current techniques allow statistical inference of functional couplings in the network but are fundamentally unable to correctly identify indirect and apparent connections between neurons, generating redundant maps with limited ability to model the causal dynamics of the network. In this paper, we describe a novel mathematically rigorous, model-free method to map effective—direct and causal—connectivity of neuronal networks from multi-electrode array data. The inference algorithm uses a combination of statistical and deterministic indicators which, first, enables identification of all existing functional links in the network and then reconstructs the directed and causal connection diagram via a super-selective rule enabling highly accurate classification of direct, indirect, and apparent links. Our method can be generally applied to the functional characterization of any *in vitro* neuronal networks. Here, we show that, given its accuracy, it can offer important insights into the functional development of *in vitro* hiPSC-derived neuronal cultures.

## 1. Introduction

*In vitro* cultures of primary neurons can self-organize into networks that generate spontaneous patterns of activity (Segev et al., 2001; Wagenaar et al., 2006; Chiappalone et al., 2007), in some cases resembling aspects of developing brain circuits (Gutnick et al., 1982; Meister et al., 1991). The emergent functional states exhibited by these neuronal ensembles have been the focus of attention for many years (Eckmann et al., 2007; Yuste, 2015) as they can be used to investigate principles that govern their development and maintenance (Marom and Shahaf, 2002; Opitz et al., 2002) and to produce biological correlates for neural network modeling (Churchland and Sejnowski, 1992; Sporns et al., 2005). The introduction of human induced-pluripotent stem cell (hiPSC) technologies (Takahashi et al., 2007; Yu et al., 2007) opened the possibility to generate *in vitro* neuronal networks in neurotypical (Odawara et al., 2014; Kirwan et al., 2015), as well as patient-specific genetic backgrounds (Brennand et al., 2011; Wainger et al., 2014; Woodard et al., 2014; Canals et al., 2015; Nageshappa et al., 2015; Sarkar et al., 2018), demonstrating the potential to reproduce key molecular and pathophysiological processes in highly controlled, reduced, experimental models that enable the study of neurological disorders and the discovery and testing of drugs, especially in the context of the individual patient (Trujillo et al., 2016; Fink and Levine, 2018; Silva and Haggarty, 2019).

One common approach to obtain information from *in vitro* neuronal networks is to record their activity via multi-electrode array (MEA) or calcium fluorescence imaging and then use network activity features to describe their physiology. One main limitation, however, is that these high-dimensional data, which report about the information representation in the network, do not translate into a clear understanding of how this representation was produced and how it emerged based on neuronal connectivity (de Abril et al., 2018). The synchronization of spontaneous spike trains among different MEA sites or neurons, also referred to as network bursting, is an example of observed neural behaviors widely reported in the literature. The generation of network bursting in an *in vitro* neuronal culture is evidence that the neurons are synaptically connected. However, the extracellular nature of the MEA recording does not provide information about how neurons are connected and how signals propagate between them, such that computational analyses are necessary to reconstruct their complex dynamic patterns and relate their emergence to the underlying wiring diagram (Sporns et al., 2005). However, this kind of analysis presents several challenges as it requires not only identification of functional relationships between cells, but also reconstruction of the dynamic causality (i.e., the knowledge of which neuron fires first and affects another one) between directly linked neurons that are simultaneously involved in several different signaling pathways. This defines the difference between functional and effective connectivity inference: the first only reports about statistical dependencies between cells' activities without giving any information about specific causal and direct effects existing between two neurons (Wang et al., 2014); the second attempts to capture a network of effective—direct and causal—effects between neural elements (Sporns, 2013b).

Model-based approaches have been proposed for inference of effective connectivity (Makarov et al., 2005; de Abril et al., 2018). Among them, dynamic causal modeling (DCM) (Friston et al., 2003) and structural equation modeling (McLntosh and Gonzalez-Lima, 1994) variants have shown best performances. Model-based methods grounded in Ising-like models, which include maximum entropy inference (Schneidman et al., 2006) and maximum likelihood variants [“kinetic Ising models” or, more generally, generalized linear models (GLMs)] (Hertz et al., 2011; Roudi et al., 2015), are also worth mentioning. However, these methods estimate the effective connectivity of a measured neuronal network by explicitly modeling the data generation process, i.e., only the connectivity of a simulated network model is inferred without any theoretical guarantee about its accuracy and its ability to correctly estimate the connectivity of the biological network (Wang et al., 2014; de Abril et al., 2018).

**Figure 1:**
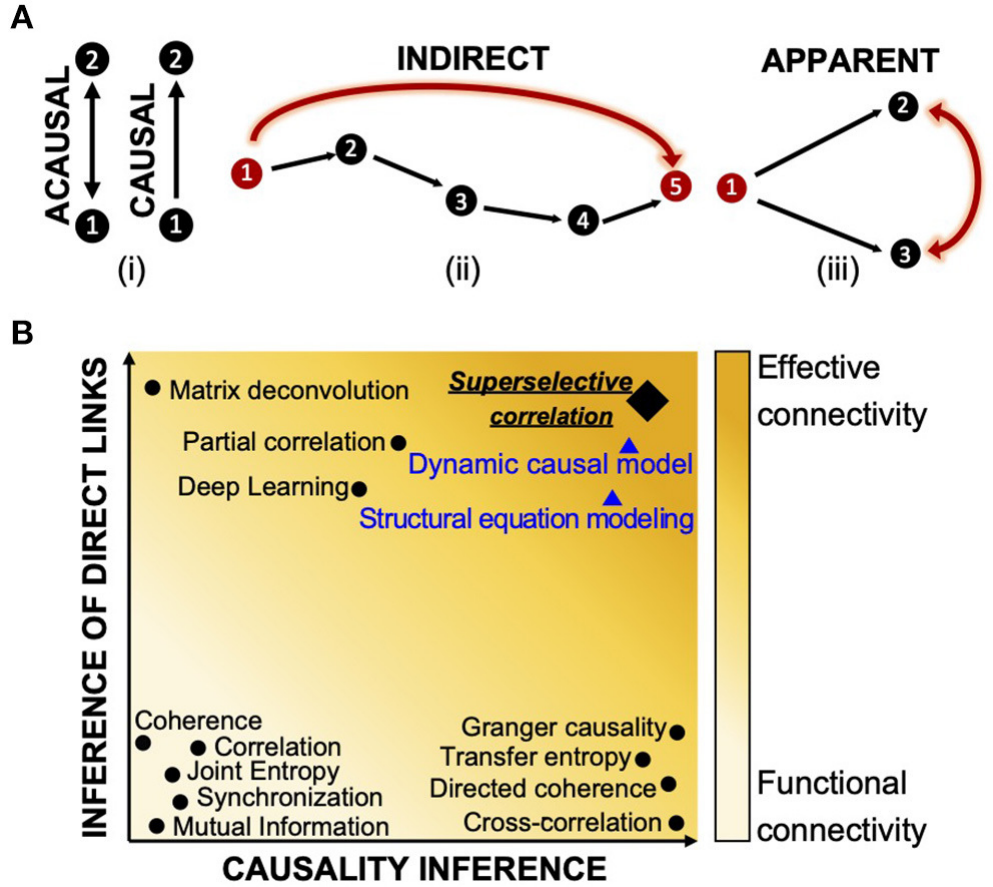
Definition of functional and effective connectivity. **(A)** Classification of causal (directional link), indirect (multi-neurons pathway), and apparent (functional coupling due to common input) connectivity. **(B)** Classification of most common connectivity inference methods in terms of causality and detection of direct links. On the *x*-axes, the graph shows a scale of causality that refers to the ability of a given connectivity method to infer or not the directionality of the functional connections between neurons. On the *y*-axes, the graph visually quantifies the capabilities of one approach to detect direct links between neurons by identifying and discarding multi-neurons connections and apparent ones. Indicators that are acausal and do not infer direct links can only report about functional connectivity (light yellow); indicators which contain information about direction of interaction and direct neuron-to-neuron communication are close to the inference of effective connectivity (orange color). Most common model-free techniques are indicated with black dots. Model-based methods are reported with blue triangles and their ability to infer effective connectivity is negatively weighted by the impossibility to test their performances. The super-selective correlation approach we propose is reported in black like the other model-free methods; a diamond signal is used to emphasize the fact that, although being model-free, it aims at inferring effective connections. The graph visually summarizes results of comparisons between connectivity methods from review papers (Garofalo et al., 2009; Wang et al., 2014; de Abril et al., 2018) and do not contain precise quantitative information about the differences.

Because of this limitation, descriptive, model-free approaches are usually preferred as they are easy to implement, rely on a limited number of assumptions that are directly related to the investigated neuronal network, and can be more easily validated (Makarov et al., 2005; de Abril et al., 2018). A number of model-free methods proposed for reconstructing the connectivity of *in vitro* neuronal networks (Garofalo et al., 2009) have been previously reviewed (Pereda et al., 2005) and tested (Wang et al., 2014). However, because they rely on purely statistical indicators, they can only infer how neurons are functionally coupled, but lack the ability to identify the network of effective interactions between neurons by either missing the directionality or confounding indirect and apparent links from direct ones. Directionality conveys the causality of signaling in the network, i.e., which neural element has causal influences over another (Figure 1Ai). However, causality does not imply a direct connection between two neurons. In fact, a functional coupling between two neurons can be causal even though the two neurons are not directly connected, and this may occur if there is a multi-neurons pathway between the two cells (indirect connection, Figure 1Aii), or if the connection detected between the two neurons is simply a mathematical artifact resulting from the correlation of correlations generated by common inputs from other participating neurons (apparent connection, Figure 1Aiii) (Friston, 2011).

The whole issue of estimating the network connectivity from correlation has a long history (Eggermont, 1990). Methods such as correlation (Rodgers and Nicewander, 1988), coherence (Hinich and Clay, 1968; da Silva et al., 1989; Grinsted et al., 2004), mutual information (Grassberger et al., 1991; Quiroga et al., 2002; Garofalo et al., 2009), phase and generalized synchronization (Quiroga et al., 2002; Bastos and Schoffelen, 2016), and joint entropy (Garofalo et al., 2009) describe only statistical dependencies between recorded neurons without carrying any information of causality or discriminating direct and indirect effects. Techniques such as cross-correlation (Garofalo et al., 2009; Ito et al., 2011), directed and partial directed coherence (Saito and Harashima, 1981; Baccalá and Sameshima, 2001), and Granger causality (Granger, 1969; Seth, 2010) are examples of causal indicators as they provide inference of directionality of dependence between time series based on time or phase shifts, or prediction measures. However, because these operators rely only on pairwise statistical comparisons and treats pairs of neurons independently, they show the same limitations when dealing with indirect connections and external inputs. Although the literature includes a long list of attempts in studying and addressing the ever-existing problem of inference of causation and existence of physical synaptic connections between neurons (Pernice and Rotter, 2013; Terada et al., 2020), only a few techniques competed in the challenge of inferring the effective connectivity of a network (de Abril et al., 2018). Transfer entropy (TE) is a well-known method that allows inference of effective connectivity (Vicente et al., 2010). With this, Orlandi *et al*. (Orlandi et al., 2014) proved successful in detecting the effective connectivity from simulated calcium imaging recordings and the same algorithm revealed interesting connectivity aspects in *in vitro* networks (Tibau et al., 2020). Partial-correlation (Garofalo et al., 2009; Sutera et al., 2017), which takes into account all neurons in the network, showed best performance in detecting direct associations between neurons and filtering out spurious ones (Orlandi et al., 2017). The most significant limitation of this solution is its high computational cost. Moreover, as the partial correlation matrix is symmetric, this method is not useful for detecting the causal direction of neuronal links. It also does not attempt to infer self-connections (de Abril et al., 2018). A combination of correlation and network deconvolution was used by Magrans and Nowe (de Abril and Nowe, 2015) to infer a network of undirected connections with elimination of arbitrary path lengths caused by indirect effects. However, this method also cannot identify directions of connections and the singular value decomposition of network deconvolution has an extremely high computational complexity (Orlandi et al., 2017). A convolutional neural network approach (Romaszko, 2017) showed the same limitations in computational complexity and undetected self- and causal connections. Figure 1B graphically summarizes the inference capabilities of the state-of-the-art connectivity methods as reported in Friston (2011); Wang et al. (2014); Bastos and Schoffelen (2016); de Abril et al. (2018).

**Figure 2:**
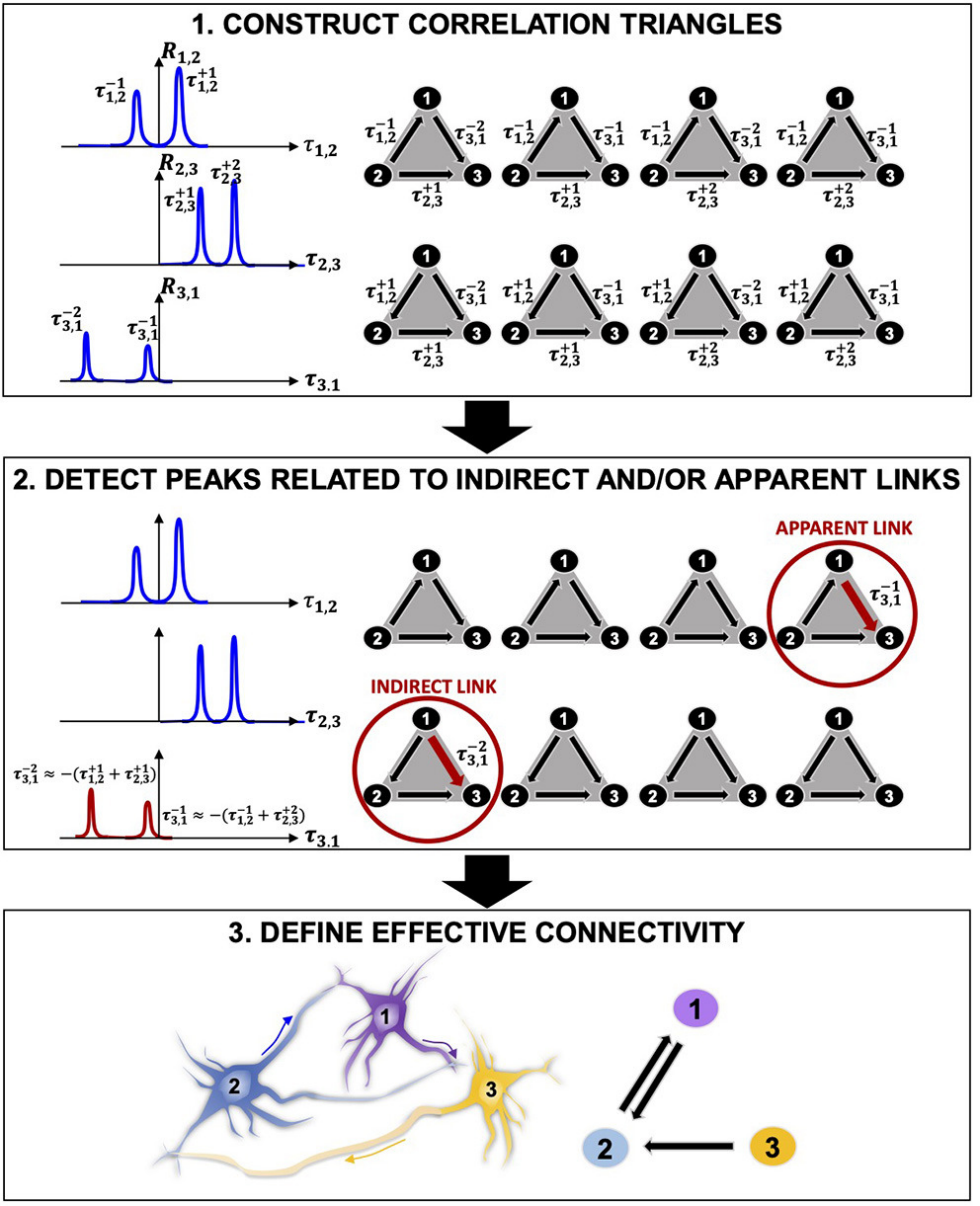
Connectivity reconstruction via detection of correlation triangles and classification of indirect and apparent links. **(1)** Given three neurons 1, 2, and 3, our algorithm searches, in time, for all correlations between them by computing the pairwise correlation functions *R*_1,2_, *R*_2,3_, and *R*_3,1_. In this representative example, the algorithm detects two correlation peaks for each pair of neurons and associates the corresponding delays of interactions ($\tau_{1,2}^{-1}$, $\tau_{1,2}^{+1}$, $\tau_{2,3}^{-1}$, $\tau_{2,3}^{-2}$, $\tau_{3,1}^{+1}$, $\tau_{3,1}^{+2}$), which are defined by the location of the peaks with respect to the origin of the *x* axis. Eight possible combinations of interactions can occur in time between the three pairs of neurons. These correspond to the eight correlation triangles shown on the right side of the panel. **(2)** Among the correlation triangles, the algorithm detects 2 critical cases ($|\tau_{1,2}^{-1}+\tau_{2,3}^{-2}+\tau_{3,1}^{+1}|<\epsilon$ and $|\tau_{1,2}^{+1}+\tau_{2,3}^{-1}+\tau_{3,1}^{+2}|<\epsilon$) and identifies the peaks relative to an indirect (multi-neurons path) and an apparent (common output) connection by searching for the ones with smaller amplitude. The smallest peaks ($\tau_{3,1}^{+1}$ and $\tau_{3,1}^{+2}$) are discarded from the analysis. **(3)** The correlation triangles are functional to the estimation of the direct connections in the network. Because no more correlation exists between neuron **1** and **3**, the estimated effective connectivity includes only the direct links for (1, 2) and (3, 1): two connections with opposite directionality exist between neuron 1 and 2 because positive and negative correlation peaks are detected in *R*_1,2_ ($\tau_{1,2}^{-1}$ and $\tau_{1,2}^{+1}$); one link connects neuron 2 to neuron 3 as a result of the positive correlation peaks in *R*_2,3_ ($\tau_{3,1}^{+1}$ and $\tau_{3,1}^{+2}$).

In this work, we propose a novel, mathematically rigorous method that uses a model-free approach (i.e., does not depend on a set of underlying assumptions about the biology of participating cells) to decompose the complex neural activity of a network into a set of numerically validated direct, causal dependencies between the active component neurons that make up the network. First, the inference power of statistical approaches (signal-, network-, and information theory-based) allows mapping the functional connectivity of the network. Then, we propose a mathematically rigorous selection scheme that distinguishes between apparent or non-direct links and direct ones, therefore enabling inference of direct causal relationships between connected neurons that more realistically describe the effective connectivity of the network (Figure 2).

We evaluate the performances of the proposed method on synthetic datasets generated through simulation of an Izhikevic neuronal network model mimicking the activity of *in vitro* cultures of neurons, and demonstrate important improvements, relative to the state-of-the-art connectivity methods, to network inference accuracy due to a deterministic component of our method capable of identifying false positive (FP) connections.

We show an experimental application of our approach to spontaneously generated *in vitro* networks of hiPSC-derived neurons cultured on MEAs providing an analysis and interpretation of the physiology not possible otherwise. We describe the temporal evolution associated with the connectivity and dynamic signaling of developing hiPSC-derived neuronal networks, including increasing synchronized activity and the formation of small numbers of hyper-connected hub-like nodes, as similarly reported by others (Canals et al., 2015; Kirwan et al., 2015). These results further support the performance quality of our approach and provide an example of how this connectivity method can be used to characterize network formation and dynamics, thus facilitating efforts to generate predictive models for neurological disease, drug discovery, and neural network modeling.

## 2. Materials and Methods

### 2.1. Theoretical Framework for Connectivity Reconstruction

The central contribution of this manuscript is in providing an innovative, computationally efficient, and easy-to-apply method for decomposing the collective firing properties stored in the electrophysiological recordings from neuronal networks on MEAs into direct (one-to-one) and causal (directional) relationships between all participating neurons. We propose a multi-phase approach that identifies and discards any correlation link that does not directly relate to a direct interaction between two cells. The core of our methodology is graphically described in Figure 2 and includes three main phases: (1) statistical, correlation-based reconstruction of functional connectivity; (2) mathematically rigorous super-selection of direct links via identification of peaks related to indirect and apparent links, and (3) reconstruction of directed causal connectivity between neurons.

1. The functional connectivity (statistical dependencies) of a network is computed via pairwise correlation studies. Functional interactions between neurons are represented by correlation peaks and their delays ***τ***. The algorithm constructs ***correlation triangles*** by considering all possible combinations of correlation delays for any possible triplet of neurons (Figure 2.1). Importantly, correlation triangles do not refer to any three-neuron physical connection, sometimes referred to as “neural triangles” in the literature (Song et al., 2005; Roxin et al., 2008). Here, we define a correlation triangle as a mathematical object that our algorithm uses to classify functional interactions based on all possible triplets of correlation delays that can be formed in the network. Therefore, correlation triangles exploit the entire signal history of neurons in order to determine the correlation peaks.
2. Correlation peaks associated with indirect or apparent links in corresponding correlation triangles are discarded from the analysis by means of a mathematical super-selection rule, which deterministically classifies the type of dependence between each triplets of neurons (Figure 2.2). The super-selection rule is formally presented later. Here, it can be summarized as follows. If a correlation triangle is made up of three correlation delays that are the combination of one another, one of the component correlation delays is either representative of an indirect link (Figure 1Aii) or of an apparent link (Figure 1Aiii); therefore, this correlation delay does not refer to an effective connection and must be discarded. When the algorithm finds a correlation triangle that satisfies this condition, it deepens into the classification of the involved correlation delays and selects the correlation peak to remove based on the peak's amplitude. For example, in Figure 2.2, the algorithm identifies an indirect link between 1 and 3 (a multi-neuron pathway), and an apparent link between 1 and 3 (correlation due to a common output). The correlation peaks corresponding to these links in the correlation triangles are discarded from the analysis. Importantly, the algorithm removes correlations from the analysis, but does not remove inferred physical connections.
3. Only when all correlation peaks between two neurons are discarded, the algorithm recognizes that a specific interaction is only apparent and deletes the corresponding connection. For example, in Figure 2.3, there is no existing connection between neurons 1 and 3. The estimated effective connectivity includes only direct links for (1, 2) and (2, 3): two connections with opposite directionality exist between neuron 1 and 2 because positive and negative correlation peaks are detected in *R*_1, 2_ ($\tau_{1,2}^{-1}$ and $\tau_{1,2}^{+1}$); one link connects neuron 2 to neuron 3 as a result of the positive correlation peaks in *R*_2, 3_ ($\tau_{2,3}^{+1}$ and $\tau_{2,3}^{+2}$).

The following sections describe the mathematical details of the developed technique. The connectivity reconstruction algorithm and associated functions were implemented in MATLAB and code is available online at https://github.com/fpuppo/ECRtools.git. In Supplementary Figure 1 (see Supplementary Material), a pseudo-code of the reconstruction algorithm is also reported.

#### 2.1.1. Reconstruction of Functional Connectivity

##### 2.1.1.1. Temporal Correlations

To identify the temporal correlations between the activity of all pairs of *n* recorded neurons *j, k* ∈ {1, …, *n*} in the network, we computed the pairwise correlation function between the corresponding signals *s*_*j*_ and *s*_*k*_

(1) $$\begin{array}{l} R_{jk}\left(\tau \right)=\int_{-\infty }^{\infty }s_{j}\left(t\right)s_{k}\left(t+\tau \right)dt \end{array}$$

In this formulation, the indexes *j* and *k* are restricted to *k* > *j* in order to avoid unnecessary calculation of auto-correlations (*j* = *k*) and explicitly calculate correlations only for *k* > *j* because, thanks to the symmetry of (1),

(2) $$\begin{array}{r} R_{jk}\left(\tau \right)=R_{kj}\left(-\tau \right). \end{array}$$

Using the fast Fourier transform (FFT) and the correlation theorem, computing correlations in Equation (1) can be efficiently performed in *O*(*S*log(*S*)) with *S* the number of samples composing the signals *s*_*j*_. In fact, if F is the FFT operator and $F^{-1}$ the corresponding inverse, the correlations can be efficiently computed as $R_{jk}\left(\tau \right)=F^{-1}\left[F\left[s_{j}\right]\cdot F\left[s_{k}\right]\right]$ (Bracewell, 1999).

Peaks of *R*_*jk*_(τ) represent correlations among neurons *j* and *k*. Their amplitude can be regarded as a measure of the level of correlation between the spikes in their registered firing activities. The higher the amplitude of a peak in *R*_*jk*_(τ) (if any), the higher the probability that there is a statistical dependency between neuron *j* and neuron *k*. However, as explained earlier in the text, the existence of a functional coupling between two neurons does not necessarily imply that there is an effective connection between them. For example, the activity of any of the two neurons can have an effect on the other through one or more interconnecting cells between them (Figure 1Aii). The location of each correlation peak with respect to the origin indicates the temporal delay $\tau_{jk}^{peak}$ between the activity of neuron *j* and neuron *k* (Supplementary Figure 2). The sign of this delay, i.e., whether the peak is found on the positive or the negative quadrant of the correlation function, defines the directionality of the interaction that, in the ideal case of a direct connection, suggests which is the pre- and post-synaptic neuron in the interaction.

##### 2.1.1.2. Peak Detection

We implemented a peak detection algorithm applied to *R*_*jk*_(τ) to identify all existing functional correlations between any pair of neurons (*j, k*) in the network and to discern the directional dependency between their spiking activities.

As part of the peak detection phase, we used a smoothing Gaussian filtering (Silverman, 1986) applied directly to *R*_*jk*_(τ) to remove high frequencies and facilitate the proper identification of correlation peaks. As introduced above, we assume that a correlation peak in *R*_*jk*_(τ) represents a potential connection between *j* and *k* and that $\tau_{jk}^{peak}$ is the signal propagation delay between them. We define a temporal range (−*T*, +*T*) over which to perform the peak search.

For a given parameterization of the Gaussian filter and a defined time window (−*T*, +*T*), the peak detection algorithm allows us to identify a list of pairwise temporal delays $\tau_{jk}^{h}$ between all pairs of neurons (*j, k*) in the observed network, with *h* ∈ {−_*h*_*n*_, …, −1, 1, …, *h*_*p*__}_*jk*_, where *h*_*n*_ is the number of peaks with $\tau_{jk}^{h}<0$ and *h*_*p*_ the number of peaks with $\tau_{jk}^{h}>0$. Peaks identified on the positive ({1, …,_*h*__*p*_}_*jk*_) or negative ({−_*h*__*n*_, …, −1}_*jk*_) side indicate whether the spiking activity of neuron *j* has temporally occurred, respectively, before ($\tau_{jk}^{h}>0$) or after ($\tau_{jk}^{h}<0$) the firing of neuron *k*. Finally, from Equation (2) the following relation holds:

(3) $$\begin{array}{r} \tau_{jk}^{h}=-\tau_{kj}^{-h}. \end{array}$$

#### 2.1.2. Detection of False Positive Connections

Correlation peaks detected in *R*_*jk*_(τ) represent any type of statistical dependence between two neurons. Peaks relative to functional dependencies due to multi-neuron connections or apparent coupling are the main cause of FPs generated in the connectivity reconstruction process and a major source of error in competing methods. To address this, we introduce a framework that identifies the effective network configuration via implementation of a deterministic super-selection rule over all the detected correlation triangles.

##### 2.1.2.1. Pure Direct Connections and Correlation Triangles

In order to identify possible dependencies between correlations, i.e., if a correlation among neurons is not direct but results from a third party correlation, we consider *cyclic* triplets of correlation delays $\left(\tau_{jk}^{h},\tau_{km}^{h^{\prime }},\tau_{mj}^{h^{\prime \prime }}\right)$. Here, cyclic means that each neuron's index appears in two ordered neuron's index pairs, once as the first index and once as the second index. This cyclicity directly implies that, if we had

(4) $$\begin{array}{r} \tau_{jk}^{h}+\tau_{km}^{h^{\prime }}+\tau_{mj}^{h^{\prime \prime }}=0, \end{array}$$

one of the three delays would result from signals that correlate through an intermediary signal as shown in Figure 2.

However, since the correlations among the neurons' firing are not Dirac deltas but rather Gaussian-like, we can define a threshold ϵ > 0 such that a weak version of (4) still holds. Therefore, Equation (4) reads

(5) $$\begin{array}{r} |\tau_{jk}^{h}+\tau_{km}^{h^{\prime }}+\tau_{mj}^{h^{\prime \prime }}|<\epsilon \end{array}$$

This equation identifies the case where there is a near-perfect match between the correlation delay of each of the three cells and represents the first step in the proposed super-selection algorithm. The definition of “near-perfect match” is based on the choice of the correlation triangle threshold ϵ. In ideal conditions, ϵ → 0 and Equation (5) reduces to Equation (4).

Finally, it is useful to define a correlation triangle as the triplet $\left(\tau_{jk}^{h},\tau_{km}^{h^{\prime }},\tau_{mj}^{h^{\prime \prime }}\right)$ of cyclic correlation delays that the algorithm detects for any pair of neurons in the network. The algorithm aims to construct *n*_*jk*_ × *n*_*km*_ × *n*_*mj*_ correlations triangles given by all possible combinations of correlation peaks of any triplet of neurons' pairs (Figure 2).

To have a complete picture of our method, we can define the directed graph *G* = (*V, E*) whose vertexes *V* are the active neurons and edges *E* are the active synaptic connections. This definition implies that our algorithm reconstructs only the connections between the neurons in the culture whose activity has been recorded, and contains no reference to the full structural connectivity of the biological network under study. Within this framework, each correlation triangle shares up to three edges with *G*, and the union *E*′ of all $\tau_{jk}^{h}$ defines the direct graph *G*′ = (*V, E*′). It follows that

(6) $$\begin{array}{r} G\subseteq G^{\prime }. \end{array}$$

*G*′ can overestimate *G* because it could include connections that are FPs (Figure 2.1). For this reason, we need to determine an efficient minimization scheme to reduce *G*′ as close as possible to *G*.

##### 2.1.2.2. Edge Covering Minimization

In order to reduce *G*′ to *G*, it is sufficient that for each correlation triangle satisfying Equation (4) [or Equation (5)], we identify the dependent $\tau_{jk}^{h}$ and remove it from *E*′. Therefore, the challenge is to find a discrimination rule that allows us to select the correct $\tau_{jk}^{h}$ in the correlation triangles that satisfy Equation (4) [or Equation (5)].

**Figure 3:**
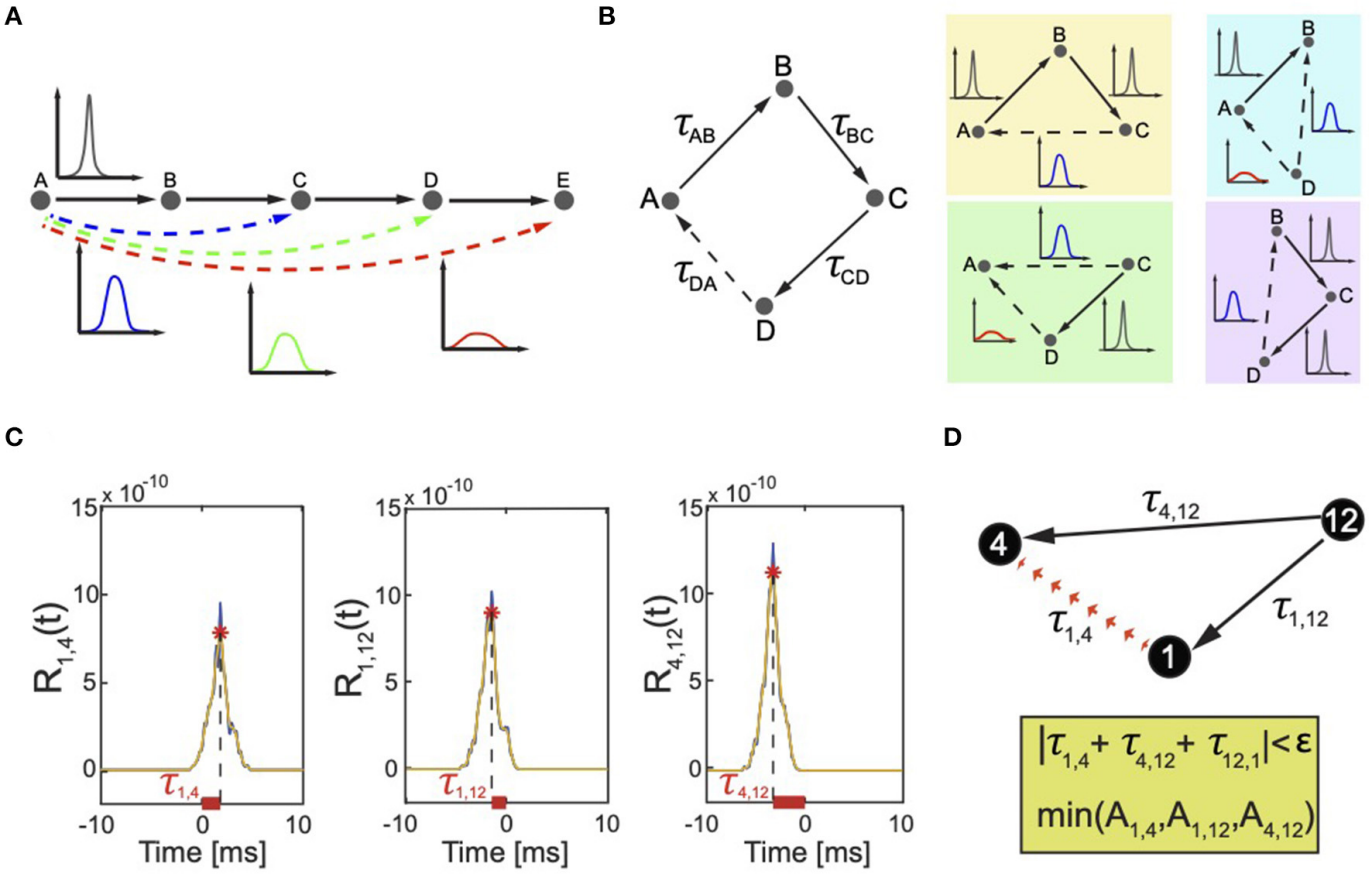
Correlation peaks and correlation triangles. **(A)** The effect of indirect connectivity on correlation peaks. The edge AB, BC, CD, and DE are directed. The associated correlation peaks are high and narrow because there is a direct dependency between the activity of neuron A (B,C,D or E) and the one of neuron B (C,D or E). The links AC, AD, and AE are indirect, with increasing order of correlation between the neurons because of more intermediary cells on the paths that link A to C, D, and E. The longer the path, the weaker the dependency between the neurons that results in a lower and wider correlation peak. **(B)** Correlation triangles reconstruct all possible dependencies between neurons. On the left, a graph composed of four neurons A, B, C, and D (polygon ABCD). Each edge is associated with a correlation peak and corresponding correlation delay τ_*AB*_, τ_*BC*_, τ_*CD*_, and τ_*DA*_. True connections are indicated by solid lines. The delay τ_*DA*_ corresponds to an indirect connection, which the algorithm must identify and discard. This is indicated by a dotted line. The edges AB and AC are apparent edges that are always present when signals are correlated and Equation (7) is satisfied. Therefore, the polygon ABCD can be decomposed in four triangles ABC, ACD, ABD, and BCD (each color represents a different triplet). The participating correlation triangles reconstruct all possible dependencies among all neurons in the polygon ABCD. Each correlation link is associated with a correlation peak whose amplitude and width depends on the length of the indirect correlation path connecting the two correlated neurons (see Figure 3A). In triangles ACD and ABD, the delay τ_*DA*_ results from a signal that has propagated through three links AB, BC, and CD. Thus, τ_*DA*_ will be detected and discarded because having lower amplitude and wider peak than the other peaks. **(C)** Real case scenario of correlation triangles and detection of FP connections. Correlation functions *R*_1,4_, *R*_1,12_, *R*_4,12_ computed for pairs of biological neurons (1, 4), (1, 12), (4, 12) (blue line) connected as in panel (B). The selected time window was (-20 ms, +20 ms), but the graph only shows a zoomed view in (−10 ms, +10 ms) to better describe the selection rule if only one correlation was detected in time between the neurons. The σ of the Gaussian filter was 0.0005 s, resulting in smoothed correlation signals (orange dashed line). The detected correlation peaks (red stars) represent functional correlations between neurons and their combinations define the correlation triangles that our algorithm uses to detect FP connections. The horizontal offset between the position of each peak and the origin represents the temporal delay τ_*j,k*_ associated with these links. **(D)** These delays satisfy the cyclic condition on τ, i.e., |τ_1, 4_ + τ_4, 12_ + τ_12, 1_| = |τ_1, 4_ + τ_4, 12_ − τ_1, 12_| < 3 ms, therefore indicating that one of the three correlations in the correlation triangle corresponds to an apparent link. The algorithm detects which of the three by searching for the correlation peak with smallest amplitude. In the example, *A*_1,4_ has the smallest amplitude and the algorithm discards it from the analysis preventing generation of an FP link.

By considering the nature of neuronal signals, we can define the discrimination factor based on the amplitude $A_{jm}^{h}$ of correlation peaks. In fact, a neuron is equivalent to an input–output object that generates an output signal *s*_*out*_(*t*) either independently or dependently on an input signal *s*_*in*_(*t*) coming from another neuron. If *s*_*out*_ depends on *s*_*in*_, the two signals are not perfectly synchronized but are often noisy (have a phase noise) resulting from the intrinsic excitability properties of the neurons. For example, when an incoming spike train from an input neuron activates an output neuron, the timing of the output spiking depends on many biochemical factors including for example the state of voltage gated ion channels. The result is that the signal of the output neuron is never triggered at the same exact delayed time, but varies. The larger the variation of the delayed timing between the input and the output neurons, the larger the phase noise in the associated correlation peak that will have smaller amplitude and larger standard deviation than the correlation among neurons with input and output signals without phase noise and perfectly synchronized. Moreover, if the signals belong to two neurons that are interconnected via intermediary cells, the phase noise is amplified and the correlation peak is even shorter (low amplitude) and wider (large standard deviation) (Figure 3A).

Our method takes into consideration only triangles because a similar analysis performed on higher degree polygons would be redundant. To clarify this point, let us consider the example reported in Figure 3B. Let us take a graph composed of four nodes. If we consider the polygon ABCD, then there will be a dependency between the edges (the correlation delays) iff

(7) $$\begin{array}{r} |\tau_{AB}+\tau_{BC}+\tau_{CD}+\tau_{CD}|<\epsilon \end{array}$$

Therefore, one of these edges is dependent and the algorithm should discard it from the analysis. Let us say that, for example, τ_*DA*_ is the indirect connection. In this case, the two additional apparent edges AB and AC are always present because signals are correlated when Equation 7 is satisfied. Therefore, the polygon ABCD can be decomposed in four triangles ABC, ACD, ABD, BCD. If we consider the triangles ACD or ABD as in Figure 3B, in both cases, the delay τ_*DA*_ is the one detected and discarded because, as explained in Figure 3A, the corresponding correlation peak has lower amplitude and larger width as it results from a signal that has propagated through three links.

Within this picture, we can establish the second step in the super-selection algorithm as:

*Given a correlation triangle (*$\tau_{jk}^{h},\tau_{km}^{h^{\prime }},\tau_{mj}^{h^{\prime \prime }}$*), the delay associated with the smallest correlation peak amplitude*

(8) $$\begin{array}{r} min\left(A_{jk}^{h},A_{km}^{h^{\prime }},A_{mj}^{h^{\prime \prime }}\right) \end{array}$$

*estimates the false positive connection and is discarded from* *E*′.

In the ideal case, i.e., without errors and approximations, this super-selection scheme eliminates all indirect correlations, and therefore reduces *G*′ exactly to *G*.

Figure 3C reports a real case scenario of apparent connection. Three neurons (indexed 1, 4, and 12 in our model) from a recorded biological neuronal network are temporally related as described by the corresponding pairwise correlations (Figure 3C) and the resulting correlation triangle schema visualized in Figure 3D. The algorithm checks all correlation triangles in the recorded network and detects that this particular case satisfies equation 5 for a selected ϵ=3 ms. As explained in the following section, ϵ was taken equal to the mean width of the correlation peaks. One correlation delay matches the combination of the other two. The algorithm identifies which of the three peaks corresponds to an indirect or apparent link by comparing the peaks' amplitude. In the example, the delay τ_1, 4_ corresponds to the smallest correlation peak and is therefore discarded from the analysis, i.e., from *E*′. If in the chosen temporal window that defines *E*′ only τ_1,4_ is detected for neuron 1 and 4, the final reconstruction will not include any effective connection between 1 and 4. This is a nice example of marrying-parents effect (de Abril et al., 2018) where an apparent link between neuron 1 and 4 is formed as a result of neuron 12 firing at the same time on both of them.

#### 2.1.3. Connectivity Matrix Reconstruction

There are three fundamental parameters that affect the inference performance of this method:

- *T* defines the time window (−*T*, +*T*) over which to search for correlation peaks in the correlation function *R*_*jk*_. This parameter affects the filtering power of the connectivity algorithm. An optimal *T* depends on the specific activity properties of the neuronal network under analysis. To account for all direct neural interactions, the best time window should include the mean maximum propagation delay between the neurons in the network. If the window is too small, the algorithm can over-filter otherwise important correlation interactions.
- σ is the standard deviation (width) of the smoothing Gaussian filter and defines the frequencies to filter out in the correlation functions. σ is important because the location of the detected correlation peaks weakly depends on it. In fact, while the Gaussian filter is necessary for a more reliable peak detection, the level of smoothing introduces a small temporal jitter between the actual location of the peak and its filtered version.
- ϵ is the correlation triangle threshold below which the correlation delays in a correlation triangle can be considered the combination of one another, i.e., how similar the combination of two correlation delays τ_*km*_ and τ_*mj*_ must be from the direct correlation delay τ_*jk*_ to estimate a three-neuron effective connectivity (see Equation 5). Large ϵ values increase the number of detected correlation peaks and computed correlation triangles with the potential shortcoming that true positive (TP) connections are filtered out. Too small ϵ values are responsible for a poor filtering of spurious delays. A good approximation for ϵ is the mean width of the detected correlation peaks which represents the variance of the correlation delays. For example, if we consider the correlation peaks from the real case scenario in Figure 3C, the width of the peaks is about 2.5 ms; therefore, we chose ϵ = 3 ms as threshold value for our super-selection of direct links.

All three parameters should be tuned in order to have the best outcome from our method.

**Figure 4:**
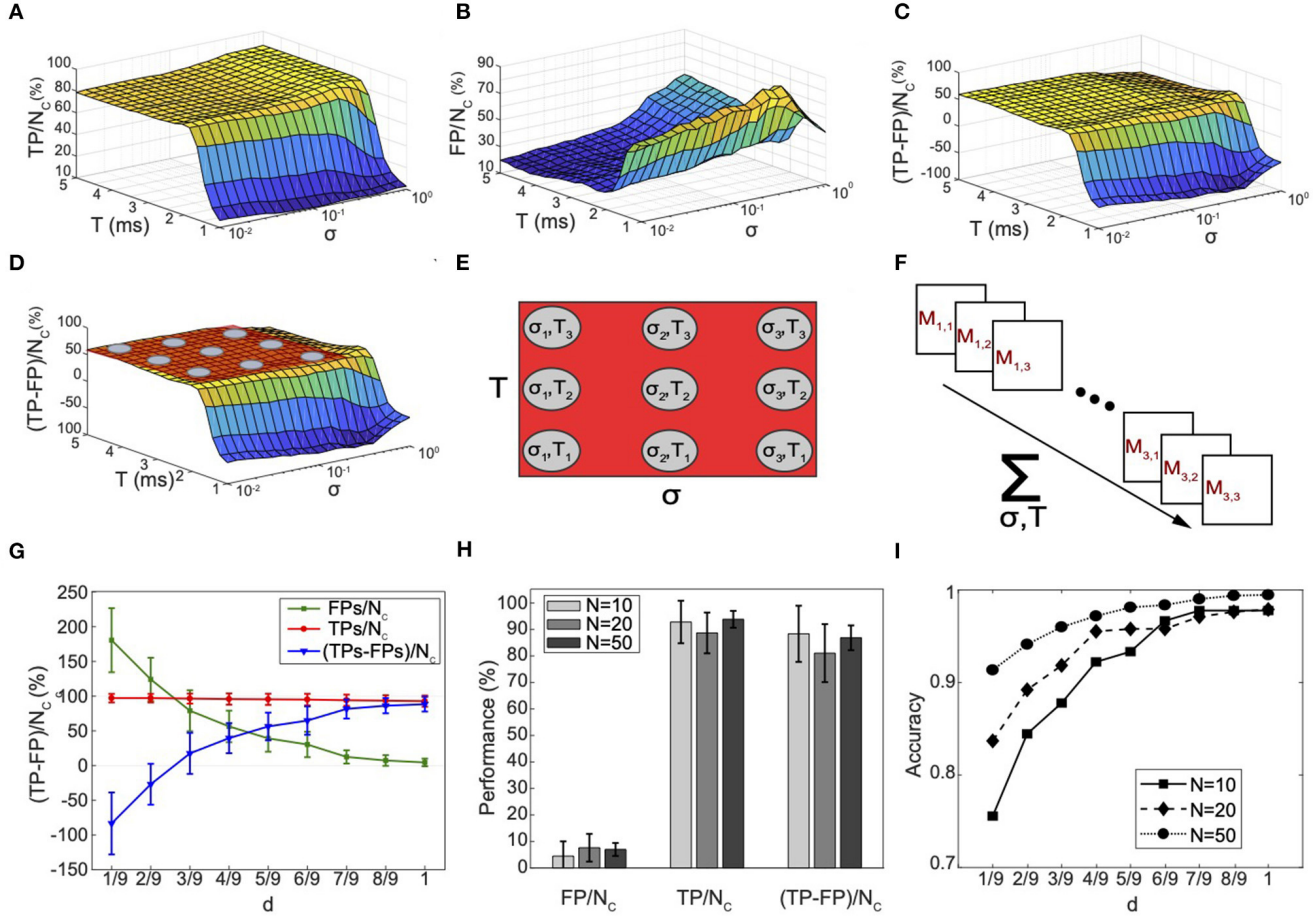
Connectivity reconstruction and performance evaluation in synthetic neuronal networks. **(A–C)** Evaluation of the performance of the connectivity method under varying time window (−*T*, +*T*), standard deviation σ, and for a fixed ϵ = 0.7 ms, for one simulated network of 10 neurons. **(A)** Net number of detected true positives *TP*/*n*_*c*_. **(B)** Net number of FPs *FP*/*n*_*c*_. **(C)** Ratio Δ = (*TP*−*FP*)/*n*_*c*_ as chosen metric for evaluation. *n*_*c*_ is the known total number of connections in the simulated network. **(D)** Visual highlight on the behavior of Δ: a peak of performance is reached around 65%; low variability is proven for a wide range of *T* and σ (plateau indicated by the red plane). **(E)** Definition of a collection of *K* = 9 points *p* in the *T*-σ space for which the algorithm shows performances falling in correspondence of the plateau area. **(F)** Abstract representation of the statistical method for recognition of FPs connections and connectivity reconstruction. For each point a connectivity matrix *M*_*p*_ is computed, resulting in the computation of *K* = 9 different connectivity matrices for the same input network. Combination of the results enables computation of the frequency *f*_*jk*_ for each connection following Equation (9). **(G)** Average (*N* = 20) performances computed at different discrimination threshold *d*. The TPs remain roughly constant. On the other hand, the FPs decrease and fluctuate around very small percentage. The algorithm filters out the FPs that fluctuate at high frequency reaching best performances (85%) at *d* = 1. **(H)** Analysis of scaling properties. An average (*N* = 20) number of *TP*/*n*_*c*_, *FP*/*n*_*c*_, and Δ was computed for 20 randomly generated networks with 10, 20, and 50 neurons, respectively. Very good performances are maintained constant for increasing network size. **(I)** Accuracy *ACC* indicator computed for different network sizes as a function of the discrimination threshold *d*. The error bars stand for the standard deviation of a dataset of 20 different networks.

The connectivity reconstruction approach we propose here is based on a parameter variation scheme that leads directly to the reconstruction of the effective connectivity matrix of the network. We used *TP*/*n*_*c*_ [net number of TP connections, with *n*_*c*_ the number of connections in the simulated neuronal network], *FP*/*n*_*c*_ (net number of FP connections) and Δ = (*TP* − *FP*)/*n*_*c*_ (confidence indicator) to investigate the effect of *T*, σ, and ϵ (Figures 4A–C). The evaluation of the minimized $G_{p}^{\prime }$ enabled identification of 80–95% of the total positive direct connections present in the model (*TP*/*n*_*c*_) in a wide range of *p* = (σ, *T*), as demonstrated by the curve plateau in Figure 4A. The remaining percentage of connections corresponded to the net number of false connections (*FP*/*n*_*c*_) that the algorithm was unable to sort out (Figure 4B), which remains very small for most parameter values. For small *T* (*T* < 2 ms), we observed a decay in sensitivity clearly due to over-filtering of true direct correlations. When doing so, the FPs first increased due to complete failure of the algorithm in recognizing connections in the small temporal range of observation and then rapidly decayed for very small *T* because no peaks could be found. This demonstrates the importance of choosing larger values both for *T* and for σ rather than smaller ones in order to avoid missing any correlation information that could negatively bias the algorithm performances. Figure 4C shows the resulting variation of Δ, which reached a peak of confidence at 65% and was maintained constant for a wide range of parameters.

This process allowed us to individuate a range of values for *T* and σ where the inference performances of the algorithm reached a maximum plateau (Figure 4D), and define the reconstruction rule of our connectivity method as follows (the numerical details are discussed in the next section).

For a fixed value of ϵ, we consider a collection of *p* points in the *T*-σ space (for example the nine points in Figures 4D,E). The boundaries for σ and *T* can be chosen according to their definitions. For example, a minimum value for σ should be related to the very high frequency in the signal while the maximum value to the frequencies in the lower middle spectrum. A minimum value of *T* should be at least as large as 2–3 times the average delay among neurons to guarantee that the relevant peak are included in the analysis. On the other hand, the maximum value for *T* can be chosen as several times (for example 5–6) the average delay among neurons in order to include more correlation triangles later used to refine the selection.

Then, for each point *p* = (*T*, σ), we compute $E_{p}^{\prime }$ and perform the super-selection to minimize $E_{p}^{\prime }$. Then, we compute the connectivity matrix *M*_*p*_ (e.g., Figure 4F) with 1 in the (*j, k*) entry if the reduced $E_{p}^{\prime }$ contains at least one $\tau_{jk}^{h}>0$, and 0 otherwise. We consider only $\tau_{jk}^{h}>0$ because, according to Equation (3), if $\tau_{jk}^{h}<0$, $E_{p}^{\prime }$ includes $\tau_{kj}^{-h}>0$ that corresponds to the same correlations.

We can therefore define the frequency for each connection in the connectivity matrix as

(9) $$\begin{array}{r} f_{jk}=\frac{1}{K}\underset{p}{\sum }M_{p_{jk}} \end{array}$$

where *K* is the total number of computed points *p*. Each frequency computed in this way is a binary classifier for a given effective connection between neurons *j* and *k*. Therefore, introducing a discrimination threshold 0 ≤ *d* ≤ 1, for each connection we can decide if it is true or false and compute the fraction of true and FP connections.

### 2.2. Experimental Methodology

#### 2.2.1. Synthetic Neuronal Network Model

To develop and validate our connectivity method, we used spiking data generated via simulations of neuronal networks based on the Izhikevich model (Izhikevich, 2003) (see Supplementary Material and Supplementary Figure 3). The original code was modified to guarantee high levels of activity in the network as well as bursting like behavior similar to that registered in our experiments (Supplementary Figure 3). We performed our analysis on sparse networks (*n*_*c*_ < < *n*_*tot*_, with *n*_*c*_ the number of connections in the simulated network and *n*_*tot*_ = *TP* + *TN* + *FP* + *FN* the total number of possible connections that can be formed given the input size of the investigated network model) that could be more easily simulated and analyzed with standard computational resources and that accurately described the sparse activity of the hiPSC-derived neuronal networks we investigated. However, it is worth noting that our model is general and not restricted to sparse connectivity.

To evaluate the scaling properties of our method, we used data generated via simulations of network models having a varying size of 10, 20, and 50 nodes. For each network size, 20 different networks were randomly generated, simulated, and then analyzed for connectivity reconstruction. For each tested network, we compared the adjacency matrix reconstructed for a frequency *f*_*jk*_ to the input connectivity matrix of the simulated model.

#### 2.2.2. Performance Measures

The performances were assessed based on the indicators *TP*/*n*_*c*_ (net number of TP connections, with *n*_*c*_ the number of connections in the simulated neuronal network), *FP*/*n*_*c*_ (net number of FP connections) and Δ = (*TP* − *FP*)/*n*_*c*_ (confidence indicator). Δ is independent on the connectivity of the network being reconstructed and, because of its definition, it better defines the level of confidence in the detection of TPs by highlighting the method capabilities in rejecting or not the FP connections. For the sake of comparison with the literature, we also used the more standard accuracy measure *ACC* = (*TP* + *TN*)/*n*_*tot*_ (Garofalo et al., 2009; Poli et al., 2016).

#### 2.2.3. Numerical Experiments

We used *TP*/*n*_*c*_, *FP*/*n*_*c*_ and Δ to investigate the effect of *T*, σ and ϵ (Figures 4A–C): from pre-selected ranges of these parameters based on observations on the simulated activity, we tuned *T*, σ, and ϵ by looking at the method's performance in selected ranges (*T* ∈ (1,5) ms, ϵ = 0.7 ms, and σ∈ (10^−2^,10^0^) ms). Most of the performance data Δ distributed to form a plateau in the *T* and σ space (Figure 4D). In this plateau region, we defined *Q* = 9 points corresponding to the combinations *C*_σ, *T*_ = (σ, *T*), with *T* =2.25 ms, 3.5, 4.5, and σ = 0.013, 0.1, 0.63 ms and we used them to reconstruct the connectivity matrix based on the threshold frequency *f*_*jk*_ as formally described in the previous section.

The network model we adopted was used to generate spiking data useful to test and develop the connectivity algorithm based on analysis of correlations. However, this model was not intended to be representative of real biological neurons and does not reproduce all the specific features of electrical recordings from *in vitro* neuronal cultures. As a result, the range of parameters selected for the simulated case did not necessarily match the one for the real case and was later adapted to the data of recorded *in vitro* neuronal networks. However, the same parameters showed very high reproducibility in repeated experiments both with simulated networks and hiPSC-derived neuronal systems. Scaling performances will be discussed later in the manuscript; however, *T*, σ, and ϵ were not affected by the network size.

#### 2.2.4. Electrophysiological Characterization of hiPSC-Derived Networks

Methods for generating cortical neurons from hiPSC, analyzing the composition of the resulting cell population, and culturing on MEA are described in the Supplementary Material. hiPSC-derived cortical neurons plated on 48-well format MEA plates were recorded every week. Recordings were acquired with the Maestro recording system and Axion Integrated Studio (Axion Biosystems). A butterworth band-pass (10–2,500 Hz) filter and adaptive threshold spike detector set to 5.5X standard deviations were applied to the raw data. Raster plots of neuronal spiking activity were generated using Axion Neural Metrics Tool, and the data were analyzed using Excel (Microsoft) and GraphPad Prism version 7.00 (GraphPad Software) (Supplementary Figures 3, 4).

**Figure 5:**
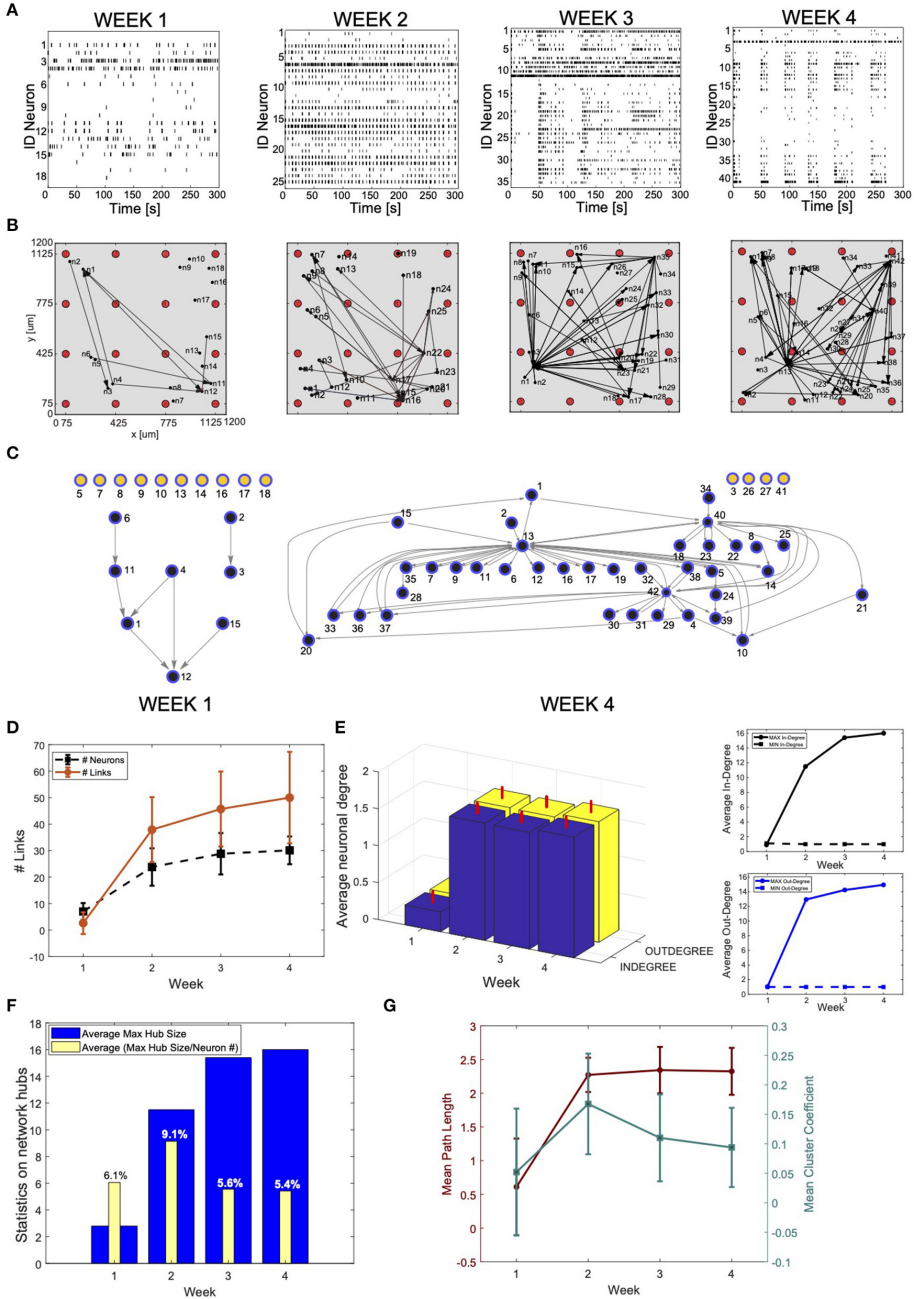
Connectivity reconstruction from neural recordings of developing induced pluripotent stem cells (hiPSC)-derived neurons. **(A)** Raster plots of the same MEA well at different time points during development: week 1, 2, 3, and 4 after plating. The four panels shows spiking signals from individual neurons (rows) obtained through spike sorting, principal component analysis (PCA), and *k*-means clustering of 300-s recordings. The culture develops complex network features: from weakly active and randomly organized (individual spiking events), to very active and fully organized (network bursts). **(B)** Estimated effective connectivity of the developing culture whose activity is described in **(A)**. Each visual map consists of a 1.2 ×1.2 mm multi-electrode array (MEA) plate (gray area), a 4-by-4 array of micro-electrodes (red circles) and the estimated directed connections (black arrows). The active neurons are represented by black dots; they are randomly distributed around their corresponding sensing electrode within a radius of 50 μm. **(C)** Directed graph relative to the culture at week 1 and 4. The connectivity is equivalent to the one visualized in B but, for clarity and consistency with the main text, links between neurons are directed edges (arrows), active neurons are network nodes (blue: connected; yellow: independent). Given a MEA well and a specific time point, indexes refer to active neurons with recorded activity reported in (A). Note that neurons mapped at week 1 do not correspond to neurons mapped in the following weeks, although they are indicated with the same indexes. **(D–G)** Graph theory based analysis of network connectivity in developing hiPSC-derived neuronal-network. **(D)** Average number (*N* = 20) of active neurons (black) and detected connections (red) in MEA wells recorded at week 1, 2, 3, and 4 after plating. The error bars represent the standard deviations for a dataset of 20 different MEA wells. **(E)** (Left panel) Statistics (*N* = 20) on the neuronal in-degree (number of input connections) (blue) and out-degree (number of output connections) (yellow). The vertical red bars stand for the standard deviations of in-degree and out-degree data. (Right panel) Average maximum (continuous line) and minimum (dashed line) in-degree (top) and out-degree (bottom). The number of in- and out- degree is equally distributed with equivalent raising behavior as a function of the cultures' developmental stage. **(F)** Statistics on network hubs as a function of the culture's age. In blue, average (*N* = 20) maximum hub size. We used the in-degree centrality measures for hub detection and characterization. In yellow, percentage of neurons that function as network hubs relative to the total number of active neurons in the well (value averaged over 20 wells). More mature networks include few super-hubs (~5% of the total number of neurons) with increase in size as demonstrated by the raising number of incoming connections at week 3 and 4. **(G)** Characterization of network segregation and integration properties. Values in the graph correspond to the mean path length (PL) and mean clustering coefficient (CCo) calculated for each well at week 1, 2, 3, and 4 after plating and then averaged over 20 wells. The error bars correspond to the standard deviation of 20 different measures. The mean PL corresponds to the average shortest path length in the networks. Infinite (absent) connections between neurons were not considered in its calculation. The PL is very low for highly immature cultures where only sparse activity from individual neurons was mainly registered. It increases as soon as connections are formed (week 2) but no longer changes with the number of new connections (week 2, 3, and 4). The cluster coefficient (CCo) is low and decreases with the maturation of the network as indication of favored segregation vs. integration with increasing number of independent but highly integrated network units (hubs).

We used routines implemented in Matlab to analyze the electrophysiological recordings and identify the active neurons in the plate. In MEA recordings, the same electrode can record the activity of multiple neurons (Supplementary Figure 6). However, identification of spikes corresponding to different neurons (Supplementary Figure 7) is crucial to interpret electrophysiological recordings, especially in connectivity studies and analyses of causal dynamics in networks (Rey et al., 2015). Therefore, we used spike sorting to group spikes with similar shape into different clusters, each corresponding to a different unit (neuron). This allowed us to isolate the activity of a few units per electrode, which resulted in the reconstruction of the activity of multiple detected neurons in the MEA well (Figure 5A). To this end, we adopted a commonly used spike sorting approach where principal component analysis (PCA) was used to extract similar features in the recorded spikes and clustering allowed us to group spikes with the same profile. We used a *k*-means clustering approach consisting of partitioning *n* observations into *k* clusters in which each observation belonged to the cluster with the nearest mean. The clustering algorithm started with a pre-defined *k* = 2. This value was then automatically updated to the best *k* estimate based on observations on the explained variance in PCA. The clustering was repeated 20 times using new initial cluster centroid positions (no change was observed for more than 20 replicates). The final output was the solution with lowest within-cluster sums of point-to-centroid distances. In a few cases, the clustering approach did not perform efficiently and generated either too many or too few clusters, which were detected by observing the increased number of outliers in the spike sorting output. In these critical cases, a visual test was performed to identify the correct clusters.

#### 2.2.5. Connectivity Analysis in hiPSC-Derived Cultures

We used our algorithm to estimate the connectivity in recorded hiPSC-derived neuronal networks at week 1, 2, 3, and 4 (Figures 5B,C). We selected a range for the parameter *T* based on considerations of the mean propagation delays between synaptically connected neurons. While physiologically many variables contribute to neuronal delays, a rough indication of the delay in synaptically connected cortical neurons was estimated to be 6–14 ms (Gonzalez-Burgos, 2000). We estimated similar conduction velocities and latency values in a previous study performed on basket and pyramidal neurons from the rat neocortex (Puppo et al., 2018). Given that monolayer networks of hiPSC-derived neurons may have temporal properties that differ from those observed in intact brain networks, we decided to avoid neglecting correlations that could negatively bias the inference performance by considering a larger temporal window (−22, 22) ms. The reconstruction analysis was then performed with *T*∈15, 22 ms to include the propagation delays measured in cortical neurons (6–14 ms) and over-estimated values to limit over-filtering. For σ, observations on the recordings and the level of smoothing required for high-frequency noise removal led us to select σ ∈ (0.3, 0.8) ms. We considered a fixed value of 3 ms equal to the average width of the correlation peaks. Within this range of parameters, we then selected *Q* = 9 points corresponding to the combinations *C*_σ, *T*_ = (σ, *T*), with *T* = 20, 17.5, 16 ms, and σ = 0.4, 0.55, 0.7 ms.

We used a graph-based connectivity analysis to estimate developing connections between neurons (Figures 5D–G). A common graph theory approach to measure the level of connectivity in a network is to investigate its centrality, which can be described as the capacity of a node to influence, or be influenced by, other system elements by virtue of its connection topology (Oldham and Fornito, 2019). The simplest and most commonly used measure of centrality is node degree, which is the number of connections attached to a node. We calculated the average in-degree and out-degree of the vertexes of each directed graph corresponding to a reconstructed network by calculating all incoming and outgoing connections for each network vertex and then averaging over the total number of vertexes per network. The statistics was then extended to 20 different wells. A node scoring highly on a given centrality measure can be considered a hub. Here, we quantified the number of network hubs at each time point (from 1st to 4th week) by computing the centrality of the graph and ranking the vertexes based on the number of incoming connections. The most important vertexes were defined network hubs. We averaged this number over 20 reconstructed networks. Finally, we studied the integration and segregation properties of the networks (Sporns, 2013a) by computing the average mean path length and mean cluster coefficient (*CCo*). We calculated the mean path length as the shortest path distance of all vertex pairs. Infinite (absent) connections between neurons were not considered in the calculation. With the use of Matlab routines, we also computed the *CCo* as the fraction of triangles around a node, which is equivalent to the fraction of node's neighbors that are neighbors of each other (Newman, 2003).

#### 2.2.6. Estimate of Signaling Probability in MEA Data

We explored the functional role of the active neurons identified in the reconstructed effective network by computing a possible estimation of their probability to be nodes “initiator,” “propagator,” or “receiver.” For a given network, we considered the neuronal activities visualized in the raster plots (Figure 5A) and inferred the behavior of each neuron by exploring the possible causality of spiking events. In brief, given a network with *n* active neurons, for each neuron *j* we counted the number of spikes *s*^+^ and *s*^−^ in the activity of all other *n*−1 neurons happening within 15 ms on either the right (*s*^+^) or left (*s*^−^) side of each spike of neuron *j*. We used *s*^−^/(*s*^+^ + *s*^−^) as an estimate of the probability that neuron *j* is a receiver. In fact, for *s*^−^/(*s*^+^+*s*^−^) close to 1 the neuron has large probability to be receiver, for values close to 0 *j* has large probability to be initiator and for values in the middle it has large probability to be propagator. We then compared this probability with the number of connections in the inferred effective connectome.

## 3. Results

### 3.1. Numerical Results

To evaluate the performances of our method, we built a numerical model by designing and simulating an Izhikevic network model mimicking the activity of *in vitro* cultures of neurons (see Supplementary Material). We studied *TP*/*n*_*c*_, *FP*/*n*_*c*_, and Δ as function of the discrimination threshold *d* (Equation 9) (Figure 4G). We note that, at a high discrimination threshold, while *TP*/*n*_*c*_ remained roughly constant (the same TPs were always detected, for all points), *FP*/*n*_*c*_ decreased toward very small percentages, smaller than the percentage corresponding to any of the *p* points. This is not surprising because the TP connections, which in most cases point to the interactions between the same pairs of neurons, were always observed for all points (*f*_*jk*_ ≈ 1, *f*_*jk*_ is the frequency for each connection in the connectivity matrix—see Equation 9). On the other hand, the FPs are just fluctuations of the algorithm as new FP connections pointing every time to a different pair of neurons were constantly generated by the algorithm for all points *p*. As a result, the same FP connections observed for a given pair of neurons are very unlikely detected by many different points, and at high frequency they are filtered out.

For example, for two given points *p*_1_ and *p*_2_ the algorithm detected 15% and 20% FPs, respectively; however, most of the FPs detected by point *p*_1_ did not correspond to the ones detected by point *p*_2_. On the other hand, the same two points *p*_1_ and *p*_2_ detected the same TPs. Through filtering, the TPs detected by both points are preserved, all FPs are discarded, resulting in 2% of FPs remaining, a percentage that is smaller than any percentage associated with each of the points. Importantly, this result demonstrates empirically our mathematical framework and it highlights its robustness in this example application.

Δ data showed average performances of 88.3%, 88.7%, and 86.8% for varying network sizes of 10, 20, and 50 neurons, respectively, therefore demonstrating a very reliable detection of connections and exceptional scaling properties in this example (Figure 4H). Figure 4I shows the accuracy of our method. It is interesting to observe how the accuracy data for *d*=1/9, i.e., prior to using the reconstruction algorithm based on the discriminator threshold *d* (Figure 4I, *d* = 1/9), show performances already higher than 75% for a network of 10 neurons, and even higher for larger sizes, thus exceeding the ones obtained before standard thresholding in published connectivity methods (Pastore et al., 2016). The complete reconstruction algorithm led to a computational accuracy close to 100% for all network sizes due to the further statistical pruning of FP connections (Figure 4I, *d*=1).

It is worth noting that, compared to more conventional indicators, Δ is independent of the network's connectivity. Since we made the hypothesis that each node in the network model has the same average connectivity (preserved in- and out-degree), which does not increase with the network's size, we do not expect and we are not interested in seeing connectivity dependent variations. On the contrary, other metrics, such as the receiving operating curve (ROC) or the accuracy (Poli et al., 2016), largely change with the connectivity properties of the network. For comparisons, we calculated the accuracy of the investigated network model. However, because the definition of this measure is based on the hypothesis that larger networks feature higher connectivity, our data overestimate the performances, especially for high network sizes (see the section 4 for further details on this).

### 3.2. Measures of Developing hiPSC Neural Networks *in vitro*

We tested our connectivity algorithm on networks of hiPSC-derived neurons cultured on 48-well MEA plates (*Axion Biosystem*). The activity was recorded periodically over 4 weeks (Supplementary Figure 4) to investigate network development. In parallel, immunofluorescent analyses (Supplementary Figure 5) of the composition of the neuronal population showed a mix of excitatory and inhibitory neurons, as well as astrocytes, resembling the physiological composition of an *in vivo* neural network.

The data show an increase in the level of activity as a function of the developmental phase, as well as the appearance of repetitive firing patterns and the formation of well-organized network bursts (Supplementary Figure 4).

We used our algorithm to estimate the connectivity in the recorded hiPSC-derived neural networks at week 1, 2, 3, and 4 (Figure 5B). Based on the position of each electrode, we estimated the localization of the recorded neurons. The neurons have been randomly distributed around their corresponding recording electrode, within a radius of 50 μm, as this is the expected sensitivity range of MEA electrodes. Figure 5C reports the corresponding graph-based schema of the reconstructed adjacency matrices in B for week 1 and 4, respectively. Both descriptions clearly demonstrate maturation of the network and increase in overall connectivity, as well as the formation of a few highly connected sub-networks within the culture.

To generate a more quantitative estimation of these network features, we used a graph-theory based analysis (Figures 5D–G). The larger number of active neurons and detected links in most developed networks is in accordance with the increasing levels of activity of the culture at later time points (Figure 5D). We observed that during spontaneous activity, the in- and out-degrees for different neurons were almost equally distributed among the active neurons, and showed an increasing trend as a function of the culture's age. Moreover, we observed formation of a specific network topology characterized by a connectivity highly centralized around a few super-hubs reciprocally linked to neighboring cells (large average degree), and a few remaining, poorly connected neurons (small average degree) (Figures 5E,F). These data confirm what is visually described in the connectivity maps and correlate with the network burst activity in the way the connectivity topology changes from random and unorganized, with most of the neurons isolated and poorly connected, to extremely structured and centered around a few hubs, a topology that becomes more evident with maturation. We also studied the integration and segregation properties of cultured hiPSC-derived neural networks (Figure 5G). The mean path length is fairly constant and does not change for increasing numbers of new connections. On the other hand, the mean CCo is low and decreases with the level of maturation of the network. This feature is indicative of more favored segregation vs. integration, with formation of highly integrated network units (hubs), similarly to observations reported by Livesey and colleagues (Kirwan et al., 2015) using rabies-virus-based trans-synaptic tracings of hiPSC-derived neuronal networks.

### 3.3. Correlation Between Inferred Network Degree and Signaling Probability Estimate in MEA Data

**Figure 6:**
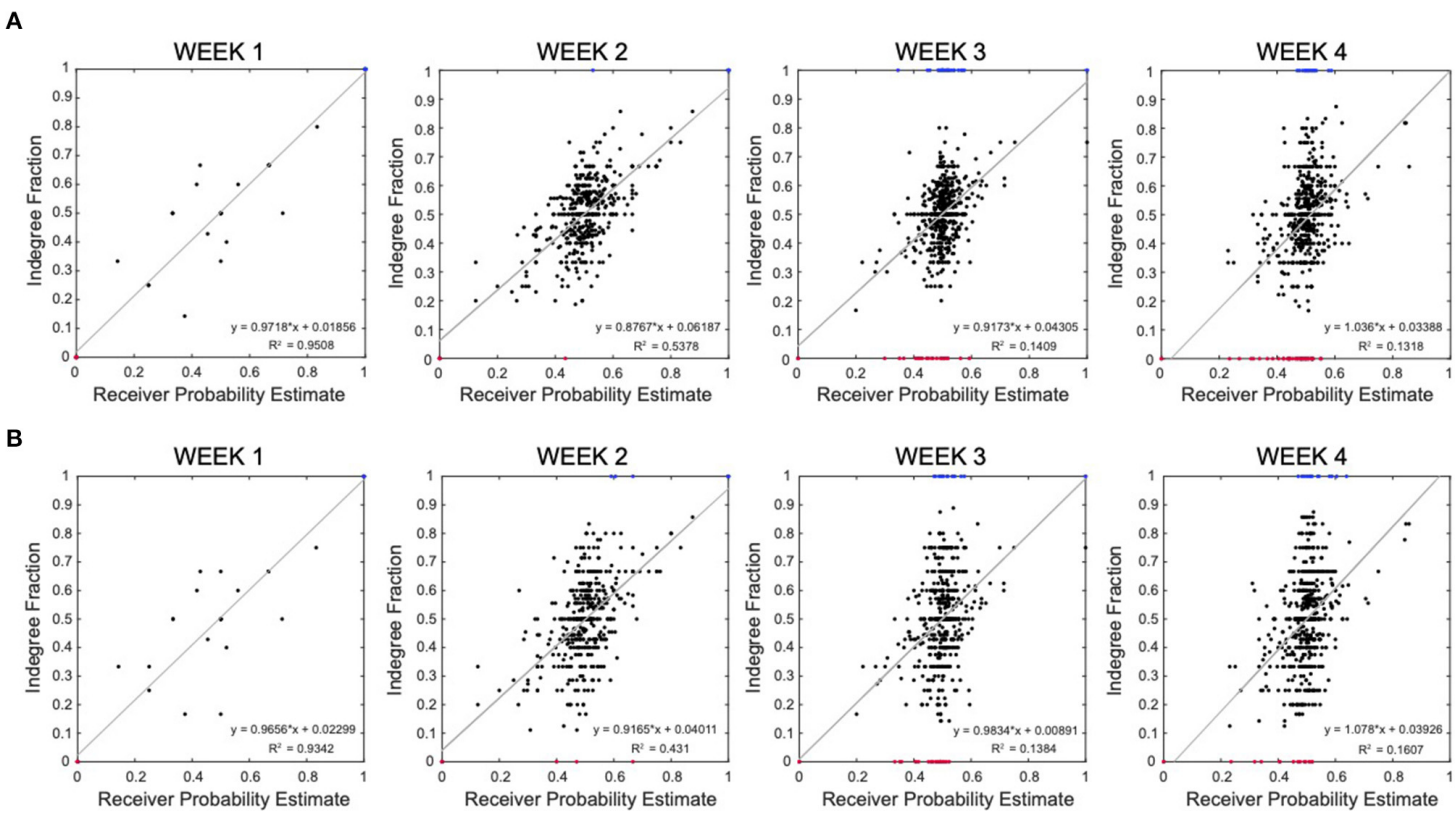
Correlation between inferred network degree and signaling probability estimate in multi-electrode array (MEA) data. **(A)** Correlation between the receiver probability estimate (x-axis) and the in-degree fraction (y-axis). Each data point in the plots represent an active neuron in a reconstructed well connectivity with computed estimate of probability of being a receiver node and in-degree fraction. The plots combine all the results from 20 wells, recorded at week 1, 2, 3, and 4, respectively. Neurons with in-degree fraction 1 (sink) and 0 (source) have been highlighted in blue and red, respectively. The gray curve is the linear regression. **(B)** Correlation between the receiver probability estimate (x-axis) and the in-degree fraction when the connectivity is inferred via correlation peak selection based on the Pearson correlation (y-axis).

We explored the functional role of the active neurons identified in the reconstructed effective network by computing a possible estimation of their probability to be nodes “initiator,” “propagator,” or “receiver” (see section 2.2.6). We then compared this probability with the number of connections in the inferred effective connectome. Figure 6 shows the correlation between the estimated probability that a neuron is a “receiver” and the computed in-degree fraction (in-degree/(in-degree + out-degree)) of that neuron in the reconstructed network. The plots combine all the results from all wells, recorded at week 1, 2, 3, and 4, respectively. Neurons with in-degree fraction 1 (sink) and 0 (source) have been highlighted in blue and red, respectively. The plots show a linear correlation between our receiver probability estimate and the reconstructed connections, which further validates the accuracy of our inference technique. This linear correlation is strong at the early stages (explained variance *R*^2^ = 0.9508 and 0.5738, at week 1 and 2) and tends to change at the later stages (explained variance *R*^2^ = 0.1409 and 0.1318, at week 3 and 4). These results suggest that our estimate of receiver probability is good and describes well the causality defined by the reconstructed connectivity when the network is less mature and the spontaneous activity is still low and non-synchronized. In this case, source (in-degree = 0) and sink (in-degree = 1) neurons are all in the corners of the plots since there is high correspondence with the receiver probability. For more developed networks expressing bursting and synchronized activity, the receiver probability estimate is no longer accurate because, due to the presence of bursts, the latency between the spikes in a given window does not capture well the direction of activation of neurons. This can be observed in Figure 6A, at week 3 and 4 where the receiver probability estimate accumulates around 0.5 while the in-degree fraction distributes across all range. This reaches the extremal discrepancy for source and sink neurons that still show receiver probability around 0.5 (red and blue data points). This is a clear fingerprint of strong bursting activity. Furthermore, it is worth highlighting that, even though the discrepancy is visually strong, the linear regression, surprisingly, still shows a near perfect 45-degree slope.

Further, we explored whether a different strategy for the calculation of the correlation peaks would change the outcome of our analysis, especially in the bursting regime which is well recognized to bias correlation studies by shadowing the causal relationships between neuron pairs (Das and Fiete, 2020). Inspired by previous works from different groups (Capone et al., 2015), we addressed this problem by adding the implementation of the Pearson correlation to calculate the correlation peaks:

(10) $$\begin{array}{r} \rho_{X,Y}=Cov\left(X,Y\right)/\sigma_{X}\sigma_{Y} \end{array}$$

This implementation can produce a different outcome of the super-selection rule especially in the presence of bursts. To test this approach, we used synthetic data from the Izhikevic model and compared the reconstruction performance with or without the use of Pearson correlation for random neuronal networks of different sizes (*n* = 10, 20, 50). For this set of synthetic data, the results (see Supplementary Figure 8 in Supplementary Material) did not show any relevant difference in accuracy and in Δ = (*TP* − *FP*)/(*n*_*c*_) mainly because, for these networks, the cross-correlation based reconstruction already reached maximum performance values that the Pearson approach could not improve further.

In Figure 6B, we repeated the analysis explained above for network reconstruction based on the Pearson correlations. The linear regressions suggest a behavior similar to the one observed in Figure 6A, with decreasing correspondence between the receiver probability and the in-degree fraction as the neuronal network turns to a bursting system. However, Pearson correlations provide more spreading along the in-degree range, suggesting a better filtering of the effect of the burst activity on the amplitude of the correlations as corroborated in literature (Das and Fiete, 2020).

## 4. Discussion

In this work, we demonstrated a new model-free based approach to infer effective—active, direct, and causal—connections from *in vitro* neuronal networks recorded on MEAs. Our algorithm offers several fundamental differences resulting in critical advancements compared to the state-of-the-art connectivity techniques, including correlation and transfer entropy variants (Pastore et al., 2016; de Abril et al., 2018). A typical challenge of model-free methods in the reconstruction of effective connectivity is to detect the causality of signaling in the network and distinguish confounding apparent connections (common input or common output) and multi-neuron pathways from direct links. Here, we defined a fundamental mathematical rule that decomposes the misleading temporal information contained in the network's temporal correlations into a set of direct, causal dependencies between the circuit's neurons via selective identification and elimination of FP connections (Figure 2).

Our method does not require any post-inference thresholding and it only depends on three fundamental parameters, *T*, σ, and ϵ, which can be easily obtained as explained in the text, and whose choice will be automated in future software release.

Evaluation of the performances on synthetic data from simulated random networks of neurons demonstrated a number of critical improvements compared to published algorithms by showing inference of causal, uni-, and bi-directional connectivity with a computational accuracy close to 1 and an excellent rate of rejection of FP connections (Figure 4).

In terms of scalability, our algorithm matches the scalability properties of most of the state-of-the-art connectivity methods which, being based on pairwise statistical and correlation studies, scale with a practical computational complexity of the order of *O*(*n*^2^), where *n* is the number of neurons. More specifically, we have three main routines whose complexity should be assessed: the computation of pairwise correlations, the peak detection algorithm, and the detection of FP connections. In order to do this, let *S* be the number of time samples of a recording for each neuron. The bare complexity of the correlation computation is *O*(*n*^2^*Slog*(*S*)) since it involves FFT evaluations. The complexity of the peak detection algorithm, on the other hand, is *O*(*n*^2^*S*). Finally, the computational complexity of the brute force detection of FP connections is *O*(*n*^3^). However, a closer look at these estimates shows that the computation of the correlation is the practical leading term. In fact, it has a larger pre-factor with respect to the peak detection, which results from the several algorithm steps included in the FFTs. Moreover, the peak detection can be restricted to just a much smaller time interval thus reducing the *O*(*n*^2^*S*) complexity to *O*(*n*^2^*S*′) with *S*′≪*S*. Finally, the computation of correlation triangles is negligible with respect to the computation of the correlations for practical cases. In fact, if we consider a standard neuronal recording, this typically has more than 10^6^ time samples. Since the computational complexity of all correlations is *O*(*n*^2^*Slog*(*S*)), unless we are dealing with more than millions of neurons, this is usually much larger than *n*^3^ three terms floating point operations needed to assess the FP connections. Moreover, with a more sophisticated approach based on dynamic programming we could compute triangles with a reduction of the *O*(*n*^3^) complexity to *O*(*n*^2^) for practical cases. However, the dynamic programming implementation details is out of the scope of this paper, even if a basic implementation is included in our software (https://github.com/fpuppo/ECRtools.git). Comparison with other techniques such as partial correlation, matrix deconvolution, or deep learning, which we found to be the only approaches able to distinguish between apparent connectivity (see Figure 1), shows usually higher computational complexity. For example, partial correlation yields a complexity of *O*(*n*^3^*S*).

Importantly, in this work we did not attempt to simulate and analyze large artificial neural networks where the number of nodes and connections aim to mimic the massive synaptic connections present in the brain. We simulated sparse networks instead, having connectivity *n*_*c*_ < *n*_*tot*_, and hypothesized average constant connectivity for larger network sizes. This simplified model was quite accurate in describing *in vitro* networks of biological neurons like the cultures of hiPSC-derived neurons we investigated (see Supplementary Figure 5). Larger networks can be simulated and tested at higher computational expenses. Based on observations on the algorithm performances, we expect approximately constant performances for increasing number of neural nodes; on the other hand, we can only speculate on the inference properties in highly connected networks (*n*_*c*_ ~ *n*_*tot*_) because of increased difficulties in properly modeling their simulated activity, as well as the anticipated increases in computational power required for processing these analyses. We expect decaying performance for networks of very high complexity due to many overlapping correlation effects from multiple cells, a problem that will require further investigation and will be addressed in future studies.

As a direct example, we have used the spontaneous firing of cultured networks of hiPSC-derived neurons to reconstruct the corresponding connectivity maps as a function of the network developmental stage (Figures 5D–G). The estimated connectivity (Figure 5) combined with quantitative analyses, such as graph-theory approaches (Figures 5D–G), enabled us to describe the developmental progress of the cultures, thus demonstrating the capability of detecting basic neuronal features such as the increased synaptic connectivity and the formation of few, highly connected network hubs. These latter are both indexes of more mature neuronal circuits and agree well with higher spiking frequencies and network burst generation observed in mature neuronal cultures (Trujillo et al., 2019).

To estimate causal and direct connections in hiPSC-derived networks, we relied on conventional spike sorting techniques in order to decompose MEA recordings into spiking activities corresponding to individual neurons. However, it is worth mentioning that despite its long history and substantial literature, spike sorting remains one of the most important and most challenging data analysis problems in neurophysiology. Spike sorting based on PCA and *k*-means clustering is one of the most accepted methods in part due to its ease of implementation. However, a number of different approaches have been proposed over the years (Caro-Martin et al., 2018). Although we recognize that the accuracy of the spike sorting procedure critically affects all subsequent analysis (Rey et al., 2015), further treatment of this issue is not trivial. Testing of performance and validation of the connectivity algorithm requires knowledge of the “ground truth” (i.e., knowing the identity of the neurons generating each detected spikes), which is only really possible in synthetic models. When applied to biological networks, our reconstruction method assumes that the spike sorting is accurate enough to separate the most prominent contributions in the activity of the network. However, the higher the accuracy of the adopted spike sorting procedure, the more precise the estimation of causal activity in the network will be. In other words, our network reconstruction methods scale with continued improvements in spike sorting. Besides, although we recognize that data sub-sampling may affect the connectivity inference (Tyrcha and Hertz, 2014; Capone et al., 2015; Huang et al., 2015), we also note that, in several studies (Capone et al., 2015), what really matters is that the inferred network is able to capture essential dynamic features of the biological one, and this is not necessarily true only when the correspondence between the inferred and the biological networks is node-by-node and link-by-link.

Furthermore, since our algorithm fully relies on neural recordings to estimate the effective connectivity of the network, the connectivity inference depends on the recording technology. MEAs devices typically available in laboratories have a low density of electrodes (typically 8 ×8 or 64 × 64 array size), which results in low spatial resolution of the sensitive area (spacing between the electrodes) and low spatial granularity in the recordings. The main consequence is reduced detection of active neurons, which affects the accuracy of reconstructing the real network's activity. This sub-sampling problem has been theoretically addressed in model-based inference techniques (Tyrcha and Hertz, 2014; Huang et al., 2015). However, in any model-free connectivity method, including the one we introduce here, the reconstruction quality can only be as good as the available measured empirical data. In our approach, unrecorded units and associated undetected correlation patterns may produce a reduction in the number of computed correlation triangles, which in turn may result in reduced filtering capabilities. As other technologies from the Brain Initiative (Litvina et al., 2019) and related efforts, such as the Human Brain Project in the EU, become available online and are validated, the methods we develop here will be in a position to take immediate advantage of them. For example, high-density MEAs (HDMEAs) that include tens of thousands of electrodes at cellular and subcellular resolution (Ullo et al., 2014; Muller et al., 2015; Yada et al., 2016; Bullmann et al., 2019) offer optimized acquisition settings and will greatly improve the resolution and accuracy of our approach. Complementary to high-resolution CMOS-MEAs, high-density arrays of individually addressable nanowires represent a viable approach to increase spatial resolution, scalability, and sensitivity of the recordings. For example, the system by Liu et al. (2017) can detect sub-threshold events, thus enabling the recording of miniature post-synaptic currents that can potentially be involved in structuring network connectivity.

Importantly, our method is scalable and can be generalized to any kind of network, thus allowing the user to target different problems in intact neurons, including synthetic models as well as *in vitro* and *in vivo* systems. Of particular relevance and as an example of an on-going effort to address these issues, our group is exploring the use of technologies to analyze electrophysiological recordings from 3D cultures. We are assessing different techniques for high-density 3D electrophysiological characterization of human-derived cortical organoids (Trujillo et al., 2019). The application of the methods we discuss here to the connectivity analysis of 3D neural structures will allow us to test a variety of hypotheses about the development of 3D neuronal networks, dynamics, and changes that may occur under external perturbation or in disease.

A limitation of the system is the inability to detect sub-threshold events due to the extracellular nature of the multi-electrode array recording, and thus to investigate the potential relationship between miniature excitatory and inhibitory post-synaptic currents and connectivity. Recently, the application of nanotechnology on neuronal electrophysiology has brought about a promising solution to overcome this limitation and to fabricate devices that are capable of detecting sub-threshold potential (Liu et al., 2017; Wei et al., 2018; Yoo et al., 2020). Although substantial engineering issues remain before the potential of nano-neural interfaces can be fully exploited (Wu et al., 2020), the future application of our algorithm to more sensitive recordings appear to be a promising approach for a more accurate understanding of network connectivity as a consequence of synaptic and extra-synaptic inputs as well as subthreshold potentials in both physiological and pathological conditions.

Finally, in this work, we have estimated the connectivity matrix of a sub-network of excitatory links, which have been described as the strongest recurrent links in the neuronal culture, major determinants of spontaneous activity (Stetter et al., 2012). However, it is increasingly clear that inhibitory connections play essential roles in neural dynamics. Therefore, further work must also be done to extend this approach to the explicit identification of inhibitory inputs and their role. Several techniques have been proposed for distinguishing the inhibitory and excitatory connections in the network (Peyrache et al., 2012; Capone et al., 2015; Pastore et al., 2018). The most direct strategy for improvement of our algorithm is by integration of cross-correlation approaches previously investigated by other groups (Pastore et al., 2018) with extension of the correlation triangle rule to account for inhibition. On the other hand, although model-based, more statistically sophisticated methods such as Ising-like approaches (Capone et al., 2015) also contain interesting elements, which could complement our analysis and facilitate an improved formulation of the inference technique.

A similar problem exists to detect excitation when there is strong tonic excitation close to saturation or persistent bursting. In the bursting regime, a functional reconstruction typically results in highly clustered connectivity due to the highly synchronized firing of large communities of neurons that appear to be all connected even though no direct synaptic connectivity exists (Stetter et al., 2012). This is a known bias associated with the “mean-field” component of the population activity that partially shadows the causal relationships between neuron pairs (Das and Fiete, 2020). In our method, the bursting regime can induce an increase in the number of computed correlation triangles with potential underestimation of FP connections due to the large number of apparent correlations between synchronized cells. However, since the Izhikevich model we used for validation includes also bursting neurons, we think that the method here proposed can already behave quite well under bursting conditions too. Nevertheless, we are currently investigating a novel approach to improve the accuracy of estimation of connectivity between bursting neurons; however, this problem remains a matter for future work.

## 5. Conclusions

We have presented an innovative approach to map the effective connectivity of neural networks from multi-electrode array data. The tool that we have developed offers critical improvements over available methods for estimating functional connectivity. Notably, our connectivity algorithm succeeds in detecting direct connections between neurons through a mathematically rigorous selection scheme that distinguishes between apparent or non-direct links and direct ones, therefore enabling inference of directed causal relationships between connected neurons. In addition, it has good scaling capabilities and can be further generalized to any kind of network, thus allowing to target different problems in intact neurons, synthetic models as well as *in vitro* and *in vivo* systems. As novel electrophysiology technologies come online and are validated, the methods we presented here will be in an immediate position to take advantage of them, resulting in fundamental improvements in spatial resolution and reconstruction accuracy. Furthermore, our algorithm can be further extended, improved, and possibly integrated with already in-use techniques to overcome important limitations such as the detection of inhibitory connections and the inference of effective connectivity in the bursting regime.

Importantly, spatiotemporal information is implicitly contained in the estimated connectivity and delay map; we expect therefore that, when used in combination with novel computational methodologies (Budd et al., 2010; Puppo et al., 2018; Silva, 2019), our method will help reveal more fundamental network properties crucial to the understanding of the relationships between network topology, dynamic signaling, and network functions in healthy and disease models.

Furthermore, it will be broadly applicable to experimental techniques for neural activation and recording, increasing its utility for the analyses of spontaneous neural activity patterns, as well as neuronal responses to pharmacological perturbations and electrical and optogenetic stimulations (Boyden et al., 2005; Hochbaum et al., 2014; Muller et al., 2015; Thunemann et al., 2018; Nguyen et al., 2019).

## Data Availability Statement

The original contributions presented in the study are included in the article/Supplementary Material, further inquiries can be directed to the corresponding author/s.

## Author Contributions

FP conceived the original hypothesis and theoretical approach, with additional contributions by GS. FP, DP, AB, and GS conceived the project. FP developed and implemented the theory, organized the study, and carried out all the computational analyses. DP and AB designed and organized the experimental studies. DP carried out all experiments and related analyses. All authors have contributed in interpreting the results and writing the manuscript.

## Conflict of Interest

The authors declare that the research was conducted in the absence of any commercial or financial relationships that could be construed as a potential conflict of interest.
