# Supplementary material for: Super-Selective Reconstruction of Causal and Direct Connectivity With Application to in vitro iPSC Neuronal Networks
Source: Front Neurosci. 2021 Jul 16;15:647877. doi: 10.3389/fnins.2021.647877 (PMC8323822; doi:10.3389/fnins.2021.647877)
Supplement: Supplementary file 1 [file Data_Sheet_1.PDF]

Supplementary Material

SUPPLEMENTARY MATERIALS

Neuronal network model

We validated the connectivity method on spiking data generated via simulations of neural networks based on the Izhikevich's model Izhikevich (2003). The original program was copied directly from his paper and modified to guarantee high levels of activity in the network as well as bursting like behavior similar to that registered in our experiments.

The voltage of each simulated cortical neuron are described by the coupled differential equations:

$$\mathbf{v}' = 0.04\mathbf{v}^2 + 5\mathbf{v} + 140 - \mathbf{u} + I_{syn} \tag{S1}$$

$$\mathbf{u}' = a(b\mathbf{v} - \mathbf{u}) \tag{S2}$$

$$\text{if } \mathbf{v}(t) = 30\text{mV, then } \mathbf{v} \leftarrow c \text{ and } \mathbf{u} \leftarrow \mathbf{u} + d \tag{S3}$$

where $\mathbf{v}$ is the neuron's voltage, $\mathbf{v}'$ is the time derivative of the voltage, **u** is a recovery variable, **u'** is the time derivative of the recovery variable, I_{syn} is the total synaptic input received by the neuron, and a, b, c and d are adjustable parameters that govern the firing behavior of the neuron. Here, the notation $\leftarrow$ indicates that the variable $\mathbf{v}$ will be assigned the value of c if the conditional statement is true. The units of time are milliseconds and the units of voltage are mV. To simulate a network of regular spiking and bursting cells, we used (a, b, c, d) = (0.02, 0.2, -65, 8).

We used the variable I_{syn} as excitatory input delivered at random times to all neurons, or a selected set of them, and simulated as a Poisson process with a mean firing rate defined *ad-hoc* for each experiment. As approximation, all neurons were considered excitatory with a homogeneous synaptic weight of 0.5 mV. Figure S.1 shows an example of random network generated via the implemented model.

Generation of in vitro neural networks of iPSC-derived neurons

All human stem cell culture was performed under approval from the Stem Cell Research Oversight (SCRO) panel at Sanford Burnham Prebys Medical Discovery Institute. Undifferentiated hiPSC Wen et al. (2014) (provided by Drs. H. Song and G. Ming, University of Pennsylvania), were cultured on irradiated mouse embryonic fibroblasts in hPSC medium (medium composition described in ref. Shi et al. (2012)). To expand, hiPSC were passaged weekly as small clusters of cells using the ethylenediaminetetraacetic acid (EDTA) solution Versene (Gibco—Thermo-Fisher Scientific) as described in Beers et al. (2012). Cortical neural progenitor cell (NPC) were differentiated from hiPSC as embryoid bodies as described in Wen et al. (2014). To differentiate to cortical neurons, NPCs were dissociated with Accutase (StemCell Technologies), plated at a density of 1 x 10^6 cells/cm^2 on a 60 mm tissue culture dish coated with 20 μg/ml laminin from Engelbreth-Holm-Swarm murine sarcoma basement membrane (Sigma-Aldrich) and then cultured for 2 weeks in neural maintenance medium (medium composition described in Shi et al. (2012)). After two weeks, neural maintenance medium was switched to a medium comprised of Neurobasal A (Gibco—Thermo-Fisher Scientific), 10% Knock-out serum replacement (Gibco—Thermo-Fisher Scientific) and 1% Penicillin streptomycin (Cellgro), and the culture was further differentiated for two more weeks. To prepare the differentiated culture for plating on imaging and MEA plates, cells were dissociated with Accutase (StemCell Technologies) for 30 minutes, strained through a 70 μm cell strainer and then re-plated at a concentration of 150,000 cells/cm^2 in Neurobasal A, 10% Knock-out serum replacement,

1% Penicillin streptomycin and 1 μg/ml of laminin on a 35 mm tissue culture dish pre-coated with 20 μg/ml laminin. After 1 day the cells were fed with the same medium plus 5 μM cytosine arabinoside (Ara C) (Sigma-Aldrich). After 2 days of Ara C exposure, the cells were plated on 48 well MEA plates (16 electrodes/well) (Axion Biosystems) previously prepared following the manufacturer's instructions, at a concentration of 75000 cells/well in a 10 μl drop. In parallel, AraC treated hiPSC derived cortical neurons were plated in 384 well imaging plates (Poly-D-lysine treated, Biocoat, Corning) coated with laminin (20 μl/well) for 1h at 37°C at a concentration of 5000 cells/well. Cells were maintained in a humidified 37°C incubator with 5% CO_2, and 66% of the medium was exchanged every other day. One week after plating the medium was changed to Brainphys medium (Stemcell Technologies).

Immunocytochemistry

Neurons on 384 well imaging plates were fixed in 4% paraformaldehyde (PFA) (Alfa Aesar Chemicals) for 10 minutes at room temperature at 2 and 4 weeks after plating. The cells were then washed 3 times in phosphate buffered saline (PBS) (Gibco—Thermo-Fisher Scientific), and then blocked in 5% donkey serum (Jackson ImmunoResearch) and permeabilzed in 0.1% Triton-X (Sigma-Aldrich) in PBS. Primary antibodies at the dilutions noted (Table S1) were incubated on the cells overnight at 4°C. The following day cells were washed 3 times in PBS, and the secondary antibodies (AlexaFluor, Molecular Probes) were added 1:500 in blocking buffer for 2 hours at room temperature. The secondary antibody was then washed several times in PBS and DAPI (Thermofisher, 1:2000 in PBS) was added for 30 minutes at room temperature. Images were acquired with the Opera Phenix High Content Screening System confocal microscope (Perkin Elmer).

TABLES AND SUPPLEMENTARY FIGURES

Antibody	Species	Dilution	Company
GABA	Rabbit	1:1000	Sigma
GFAP	Goat	1:500	Santa Cruz Biotechnologies
MAP2	Guinea Pig	1:1000	Synaptic Systems
CamKII	Rabbit	1:1000	Cell Signaling Technology
Tuj1	Mouse	1:500	Biolegend
Ctip2	Rat	1:500	Abcam

Table S1. Antibody dilutions.

Connectivity super-selection algorithm

```
# Algorithm inputs
Set: {σ}, {T}, ε
% {σ} and {T} are the collection of σ and T values for different M_p evaluation
% ε is the triangle threshold
Load: spk_wave, time  % load spike signals

# Compute correlations between neurons' pairs
R(tau) = compute_correlations(spk_wave, time, max({T}))
% R(tau) is the correlation matrix with entries R_ij(tau)
% max({T}) is the largest correlation time window considered

# Repeat for varying {σ} and {T}
for σ ← {σ}
      for T ← {T}

            p = (σ, T)
            # Process correlation peaks
            [{A^h_jk}, {tau^h_jk}] = process_peaks(R(tau), p)
            % extract amplitude and delay of correlation peaks

            # Identify correlation triangles and discard the minimum correlation
            peak for each of them
            M_p = edge_covering_minimization({A^h_jk}, {tau^h_jk}], ε)
            % apply the super-selection rule and extract direct and directed
            connections between neurons

# Reconstruct effective connectivity
f = K^-1*sum_p(M_p) % matrix of connection frequencies; K total number of points p
adjmat = f>=d  % infer the adjacency matrix selecting the connections with
frequency higher than a threshold d
```

Figure S1. **Super-selection algorithm pseudo-code.**

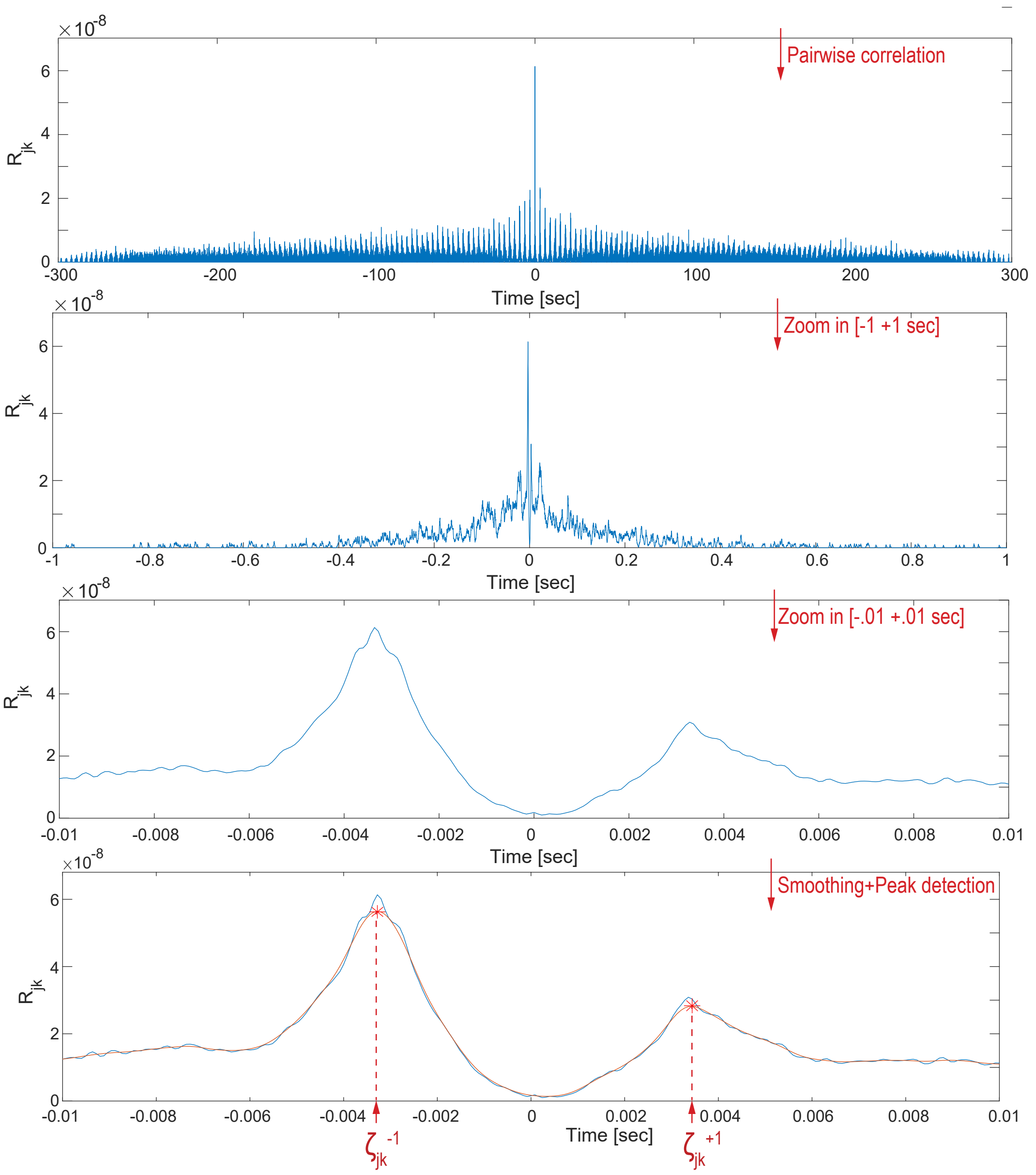

Figure S2. Correlation analysis and temporal delay detection. Correlation function computed on a pair of neurons (j, k) between their corresponding spiking signals s_j and s_k. Subsequent zooming is applied to show examples of correlation peaks. Detection of two correlation peaks is visually demonstrated in a arbitrarily defined temporal window $T = (-10, 10)$ ms. Smoothing is applied to the signal through Gaussian filtering (orange line), and then followed by peak detection on the smoothed curve. The location of each correlation peak (red stars) with respect to the origin is a first approximation prediction of the temporal delay τ_{jk}^h between the two neurons firing. Peaks detected on the positive or negative side indicate whether neuron j fires before or after neuron k and is therefore indicative of the directionality of communication flow between j nd k. The amplitude of the peak is calculated on the R_{jk} function in correspondence of the peak location.

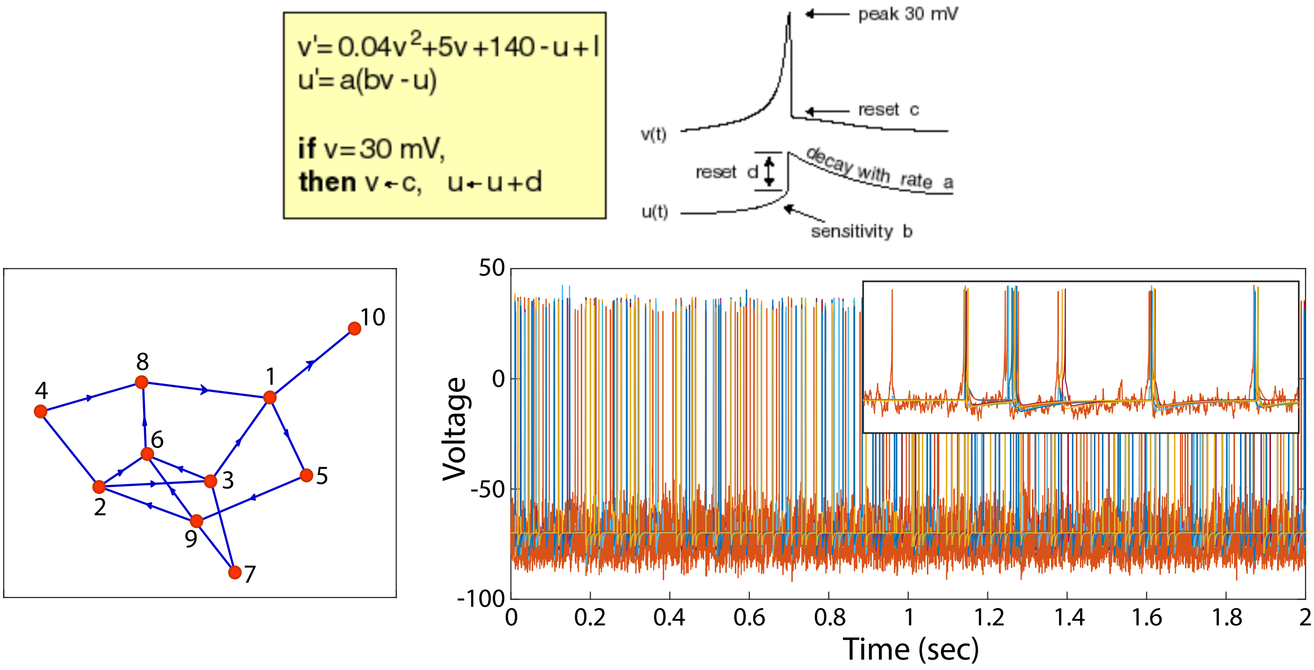

Figure S3. Neuronal network model. (A) Izhikevich model of spiking neurons. On the left, the equations of the model. On the right, visual description of the model's parameters. (reprinted from Izhikevich (2003)). (B) Directed graph (blue: edges; red: network's nodes) corresponding to an example random network of 10 neurons. (C) 20 seconds long simulation of the 10-neurons network displayed in B. Different colors stand for different active neurons. Inset: 2 sec zoomed view on the spiking activity.

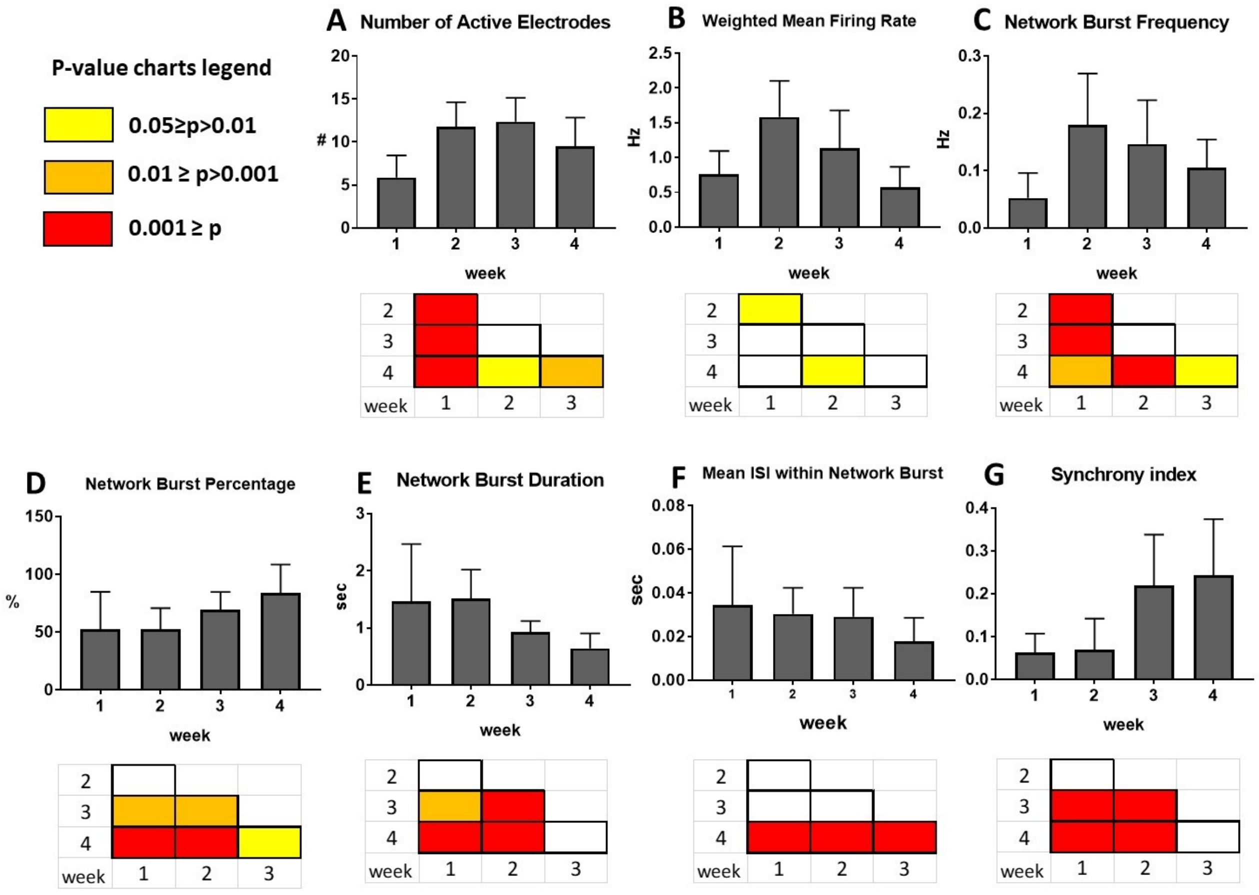

Figure S4. Development of neuronal activity and network characteristics over 4 weeks. Values are represented as average over n=24 wells and error bars indicate standard deviations. Statistical significance as heatmaps of p-values from paired t-test is shown for pairwise combinations of each week below each graph. See Methods for definition of parameters. (A) Number of Active Electrodes per well over 16 electrodes, showing a good number of electrodes recording activity over time, as well as an increase between the first and the second week. (B) The Weighted Mean Firing Rate shows an increase in neuronal activity between week 1 and week 2, while the activity decreases by week 4, as the network organization increases. (C) The Network Bursts Frequency sharply increases between week 1 and 2, when the network is formed, and then slightly decreases by week 4. (D) The Network Bursts Percentage increases over time, indicating the realization of a stable connected neural network and a reduction in sporadic extra-network firing. (E) The Network Burst Duration decreases at week 3 and 4 because of an increase in the spike frequency within a Network Burst, as shown from the decrease of Mean Inter Spike Interval (ISI) within Network Burst in panel (F), while the number of spikes per burst and the number of spikes per network burst remain constant (data not shown). (G) The Synchrony Index increases from week 2 to week 3, indicating increased participation of electrodes in synchronous network bursting.

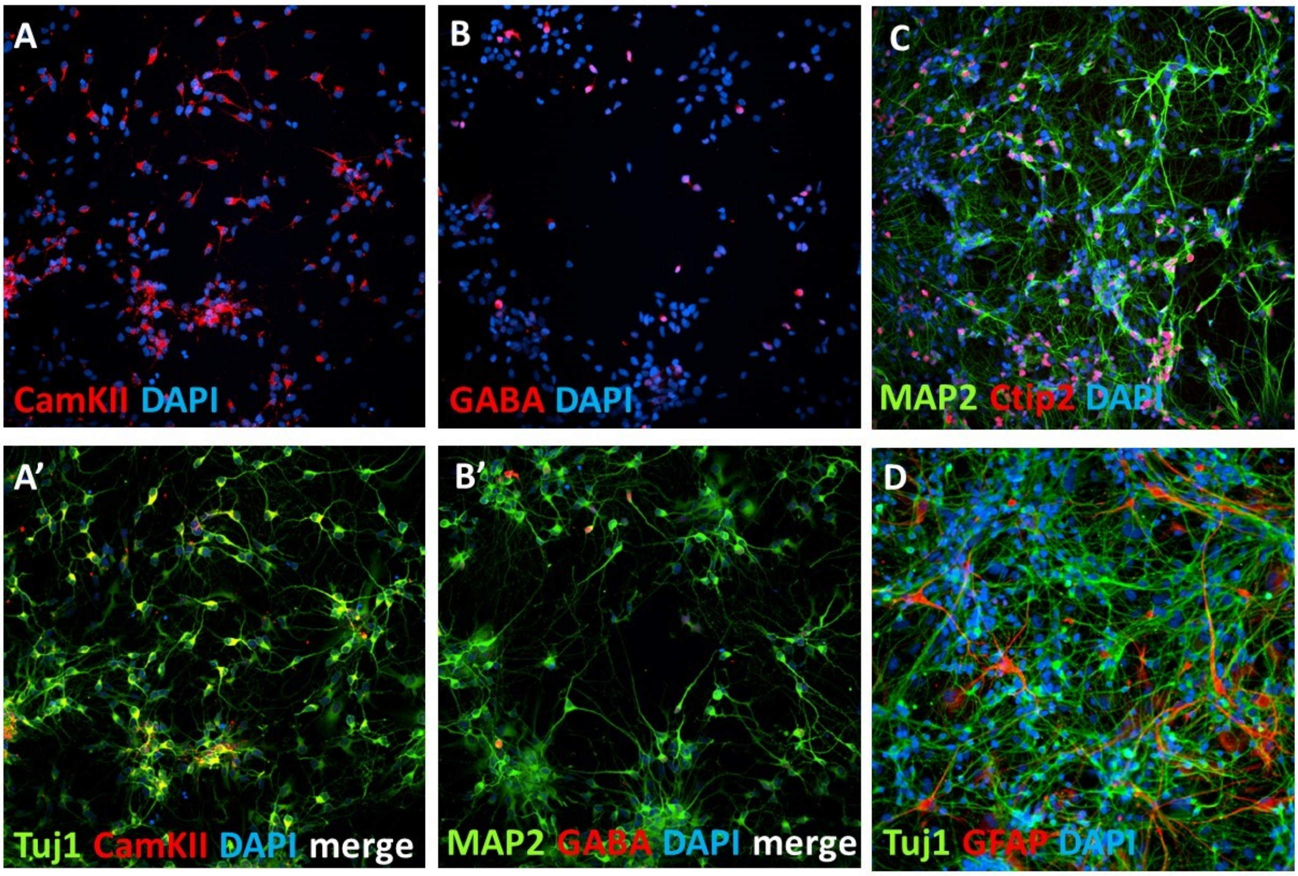

Figure S5. Characterization of human iPSC-derived cortical like neuronal culture. Neuronal culture differentiated for 4 weeks from NPC stage, re-plated on 384 well imaging plates, and then differentiated for an additional 1 week (panels A and B), 3 weeks (C) or 4 weeks (D). The percentage of positive cells are expressed as Average ± standard deviation. (A) and (A') show immunostainings for CamKII (red), a glutamatergic marker and Tuj1 (green), a neuronal marker. The percentage of TUJ1 positive neurons is 66.7%±6.8. The percentage of TUJ1 positive neurons that are co-positive for CamKII is 89.2%±6.5 (with an average of 2827±342 cells/well, n=4 wells, 9 fields per well). (B) and (B') show immunostainings for GABA (red), an inhibitory neuron marker and MAP2 (green), a dendritic marker. The percentage of TUJ1 and GABA co-positive GABAergic neurons is 8.12%±1.9 (with an average of 2377±335 cells/well, n=4 wells, 9 fields per well). (C) Immunostaining showing cells positive to CTIP2 (red), a transcriptional factor expressed by deep layer (V and VI) neurons (percentage of positive CTIP2 and MAP2 co-positive neurons is 41.7%±4.3, with an average of 3895±875 cells/well, n=4 wells, 25 fields per well). Dendritic marker MAP2 is in green. (D) Neuronal culture stained for GFAP (red), an astroglial marker, and TuJ1 (green). On an average of 4404±2727 cells per well, 27.6%±8.2 of them are positive for GFAP (n=12 wells, 25 fields per well). For all images, nuclei are stained with DAPI (blue).

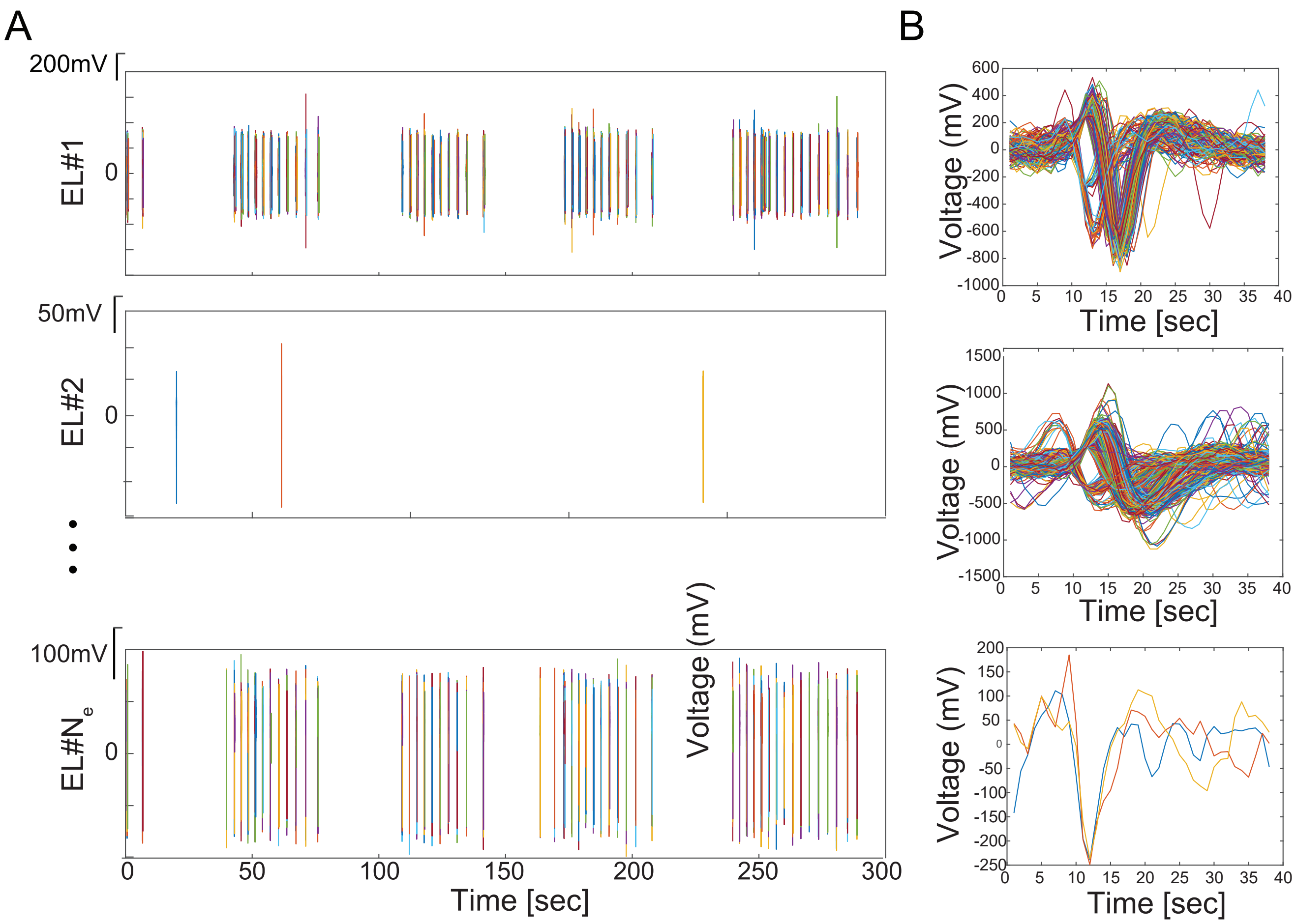

Figure S6. Examples of electrophysiological recordings from iPSC networks coupled to Multielectrode Arrays. (Left) Spike trains recorded by three different electrodes in one of the MEA's well from all detected neurons. Different colors are only used to indicate unsorted spikes in the signals. (Right) Overlaid detected spike waveforms from the corresponding signal on the left side panel.

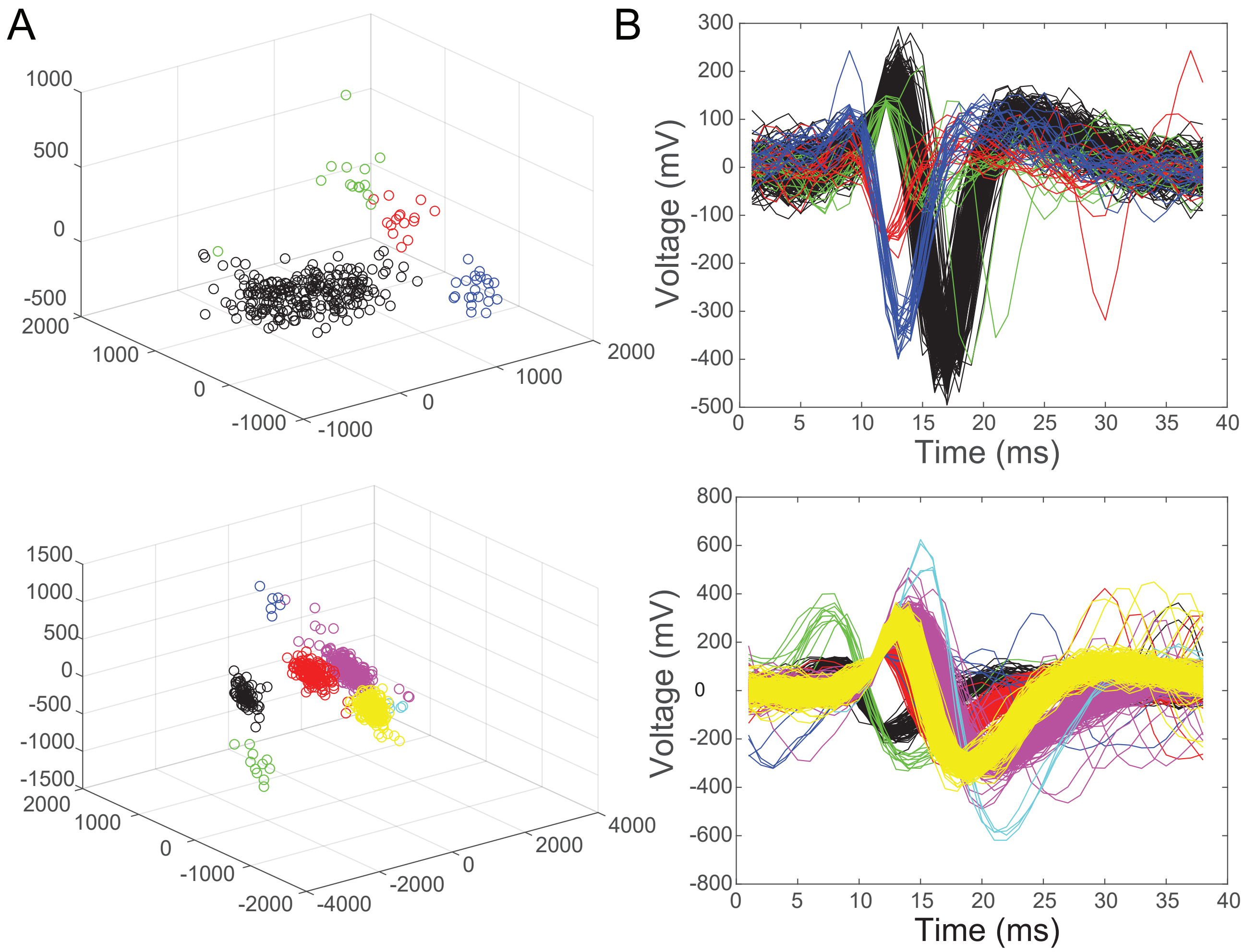

Figure S7. Examples of spike sorted data. Data were taken from the recordings reported in Figure S6 (top and middle row). PCA was used to extract similar features in the spikes' dataset. Spikes with similar features were grouped into clusters via a k-means clustering approach. This consisted of partitioning n observations into k clusters in which each observation belongs to the cluster with the nearest mean. (Left) The detected clusters after PCA analysis and k-means clustering. Different colors stand for different clusters of spike signals, each cluster belonging to a different neuron. (Right) The corresponding sorted spikes. Similar waveforms belong to the same recorded neuron.

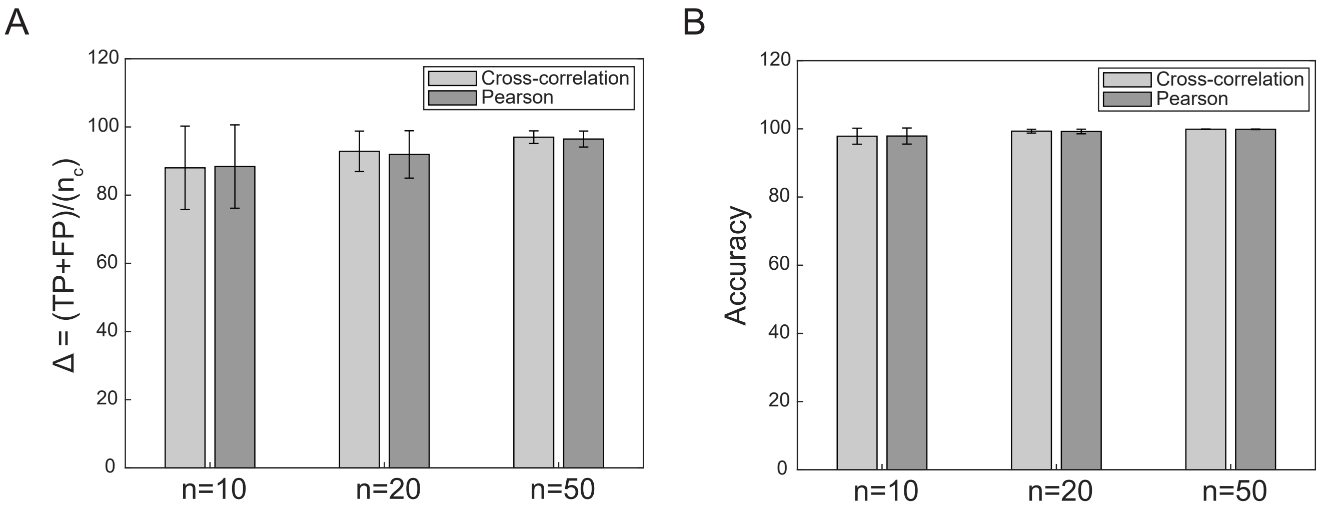

Figure S8. Connectivity inference: comparison between Cross-correlation and Pearson correlation. (A) Mean confidence indicator Δ and (B) Accuracy computed for 20 Izhikevic neuronal networks of n= 10, 20, 50 neurons, respectively, inferred via cross-correlation based or Pearson correlation based reconstruction. Error bars indicate standard deviation.

REFERENCES

Izhikevich E. Simple model of spiking neurons. *IEEE Transactions on Neural Networks* **14** (2003) 1569–1572. doi:10.1109/tnn.2003.820440.

Wen Z, Nguyen HN, Guo Z, Lalli MA, Wang X, Su Y, et al. Synaptic dysregulation in a human iPS cell model of mental disorders. *Nature* **515** (2014) 414–418. doi:10.1038/nature13716.

Shi Y, Kirwan P, Livesey FJ. Directed differentiation of human pluripotent stem cells to cerebral cortex neurons and neural networks. *Nature Protocols* **7** (2012) 1836–1846. doi:10.1038/nprot.2012.116.

Beers J, Gulbranson DR, George N, Siniscalchi LI, Jones J, Thomson JA, et al. Passaging and colony expansion of human pluripotent stem cells by enzyme-free dissociation in chemically defined culture conditions. *Nature Protocols* **7** (2012) 2029–2040. doi:10.1038/nprot.2012.130.
